# Ancestral reconstruction of mammalian FMO1 enables structural determination, revealing unique features that explain its catalytic properties

**DOI:** 10.1074/jbc.RA120.016297

**Published:** 2020-12-25

**Authors:** Gautier Bailleul, Callum R. Nicoll, María Laura Mascotti, Andrea Mattevi, Marco W. Fraaije

**Affiliations:** 1 Molecular Enzymology Group, University of Groningen, Groningen, the Netherlands; 2 Department of Biology and Biotechnology “Lazzaro Spallanzani”, University of Pavia, Pavia, Italy; 3 IMIBIO-SL CONICET, Facultad de Química Bioquímica y Farmacia, Universidad Nacional de San Luis, San Luis, Argentina

**Keywords:** flavin-containing monooxygenase (FMO), flavin adenine dinucleotide (FAD), ancestral-sequence reconstruction (ASR), NAD(P)H, enzyme kinetics, stopped flow, crystal structure, AltAncFMO, alternative versions of the ancestral flavin-containing monooxygenase, AncFMO, ancestral flavin-containing monooxygenase, ASR, ancestral-sequence reconstruction, CYP, Cytochrome P450, DDM, dodecyl-β-D-maltoside, FAD, flavin adenine dinucleotide, FMO, flavin-containing monooxygenase, hFMO, human flavin-containing monooxygenase, PP, posterior probability, T_m_, melting temperature, TRX, Triton X100

## Abstract

Mammals rely on the oxidative flavin-containing monooxygenases (FMOs) to detoxify numerous and potentially deleterious xenobiotics; this activity extends to many drugs, giving FMOs high pharmacological relevance. However, our knowledge regarding these membrane-bound enzymes has been greatly impeded by the lack of structural information. We anticipated that ancestral-sequence reconstruction could help us identify protein sequences that are more amenable to structural analysis. As such, we hereby reconstructed the mammalian ancestral protein sequences of both FMO1 and FMO4, denoted as ancestral flavin-containing monooxygenase (AncFMO)1 and AncFMO4, respectively. AncFMO1, sharing 89.5% sequence identity with human FMO1, was successfully expressed as a functional enzyme. It displayed typical FMO activities as demonstrated by oxygenating benzydamine, tamoxifen, and thioanisole, drug-related compounds known to be also accepted by human FMO1, and both NADH and NADPH cofactors could act as electron donors, a feature only described for the FMO1 paralogs. AncFMO1 crystallized as a dimer and was structurally resolved at 3.0 Å resolution. The structure harbors typical FMO aspects with the flavin adenine dinucleotide and NAD(P)H binding domains and a C-terminal transmembrane helix. Intriguingly, AncFMO1 also contains some unique features, including a significantly porous and exposed active site, and NADPH adopting a new conformation with the 2’-phosphate being pushed inside the NADP^+^ binding domain instead of being stretched out in the solvent. Overall, the ancestrally reconstructed mammalian AncFMO1 serves as the first structural model to corroborate and rationalize the catalytic properties of FMO1.

For dealing with endogenous and foreign toxic compounds, mammals and other animals developed oxidative systems that clear such potentially harmful elements from cells and tissues (1). The oxygenation of these molecules allows them to be recognized, degraded, excreted, or activated. In humans, this oxidative detoxification system is mostly based on the activity of cytochromes P450 (CYPs) monooxygenases and the flavin-containing monooxygenases (FMOs; EC 1.14.13.8). Significant advances have been made toward the characterization of human FMOs (hFMOs), but these enzymes still remain significantly less studied than CYPs. This may partly be due to the fact that CYPs are more numerous in the human proteome (57 CYPs *versus* 5 FMOs). Another factor that explains the limited insights into the functioning behind the membrane-bound hFMOs is the difficulty with which they are expressed as recombinant proteins and their challenging isolation. It is nonetheless well established that hFMOs play a crucial role in xenobiotic metabolism (2, 3).

There are 5 FMO paralogs in the human genome (FMO1-5), all clustered on chromosome 1 (4, 5). A sixth gene, called FMO6, is also present but is not translated and thus referred to as a pseudogene (6). The expression of hFMOs varies across the developmental stage and tissues. hFMO3 and hFMO5 transcripts are predominant in the liver, whereas hFMO2 is mostly found in the lung and hFMO1 in the kidney (7, 8). A good example of the pharmacological relevance of hFMOs is the role of FMO3 in the metabolic disorder called trimethylaminuria, also known as fish odor syndrome (9). Mutations in the gene encoding hFMO3 result in an inactive form of this FMO, unable to metabolize trimethylamine. The accumulation of this food ingredient in the body results in a strong unpleasant odor that may lead to social isolation and other negative effects (10).

FMOs present a tightly bound flavin adenine dinucleotide (FAD) as the prosthetic group (11, 12). Although there are many other FMOs that are not sequence-related to FMOs, this abbreviation has persisted in literature to be used to denote this specific class of detoxifying enzymes (13). The biochemical properties of mammalian FMOs were first described using enzymes from pig liver microsomes by Ziegler and Poulson in 1970 (14). In two extensive studies, Beaty and Ballou unraveled the catalytic mechanism involving the formation of a C(4α)-hydroperoxyflavin intermediate that performs the oxygenation (15, 16). Formation of this reactive enzyme intermediate is achieved by a stepwise process in which first the FAD is reduced by a hydride transfer from the NADPH (Fig. 1*A*). With the oxidized nicotinamide still bound, the reduced flavin is able to react with molecular oxygen resulting in a relatively stable C(4α)-hydroperoxyflavin intermediate (Fig. 1*B*). In the next step, when the substrate approaches the flavin, the peroxy moiety transfers an oxygen atom to a soft-nucleophilic substrate (Fig. 1*D*), whereas the other oxygen atom is released as water (Fig. 1*E*). The last step is the release of NADP^+^ (Fig. 1*F*) (17). If there is no suitable substrate bound close enough to the flavin cofactor, hydrogen peroxide is formed, which is commonly referred to as uncoupling (Fig. 1*C*).

**Figure 1:**
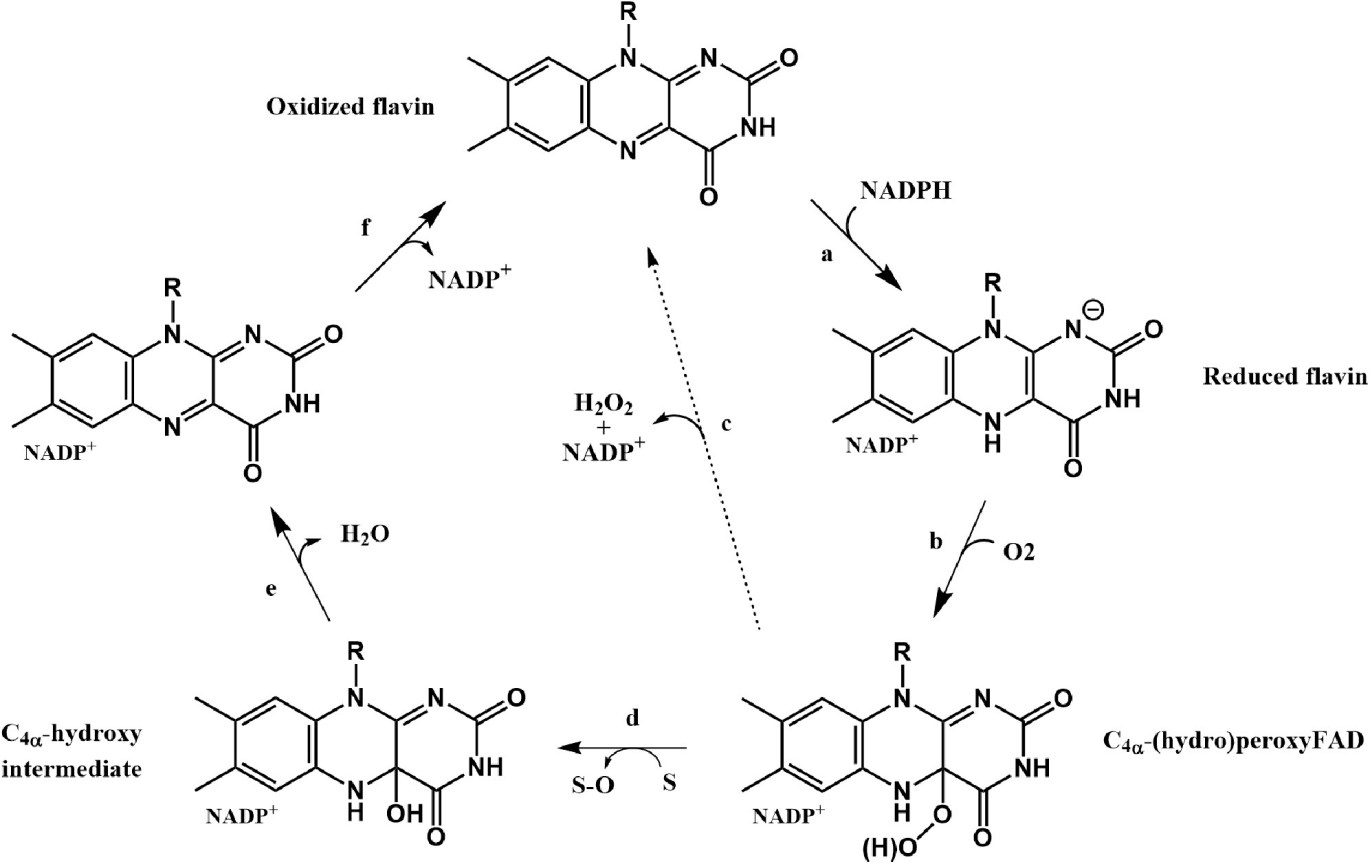
**Catalytic cycle of flavin-containing monooxygenases.***A*, binding of NADPH and subsequent reduction of the flavin. *B*, reaction with molecular oxygen and C(4α)-hydroperoxyflavin intermediate formation. *C*, uncoupling: release of hydrogen peroxide and NADP^+^. *D*, oxygen atom transfer to a substrate. *E*, release of water. *F*, release of NADP^+^.

The burst of genomic information in the last decades plus the ease of gene synthesis has enabled ancestral-sequence reconstruction (ASR) studies. ASR consists of identifying the path of changes that occur during the evolution of a specific enzyme family through the inference of the ancestors’ sequences (18). By expressing and isolating ancestrally reconstructed proteins, it is possible to follow their biochemical features or structural modifications over time in comparison with extant enzymes (19, 20). ASR has many applications for enzymologists, from the characterization of ancestral enzymes’ promiscuity or stability to the deciphering of evolutionary paths by gene duplications or the refinement of enzyme complexes (21, 22). In our previous study, using ASR, we managed to express, purify, and crystallize the mammalian ancestors of FMO2, FMO3-6, and FMO5 (23). This resulted in detailed structural insights of these resurrected FMOs. In this work, we aimed at reconstructing the ancestral mammalian FMO1 and FMO4 proteins. Surprisingly, FMO1 is one of the less studied hFMOs despite the fact that it was used as model FMO in the pioneering studies by Beaty and Ballou (15, 16). Critically, it has been established that hFMO1 displays a broad range of substrates, accepting drugs such as benzydamine, ethionamide, tamoxifen, or voriconazole (2). We present for the first time the crystal structure of AncFMO1 together with its catalytic and kinetic features. These data shed light on the different enzymatic behaviors of mammalian FMOs and will improve our understanding of their physiological roles.

## Results

### Ancestral-sequence reconstruction

**Figure 2:**
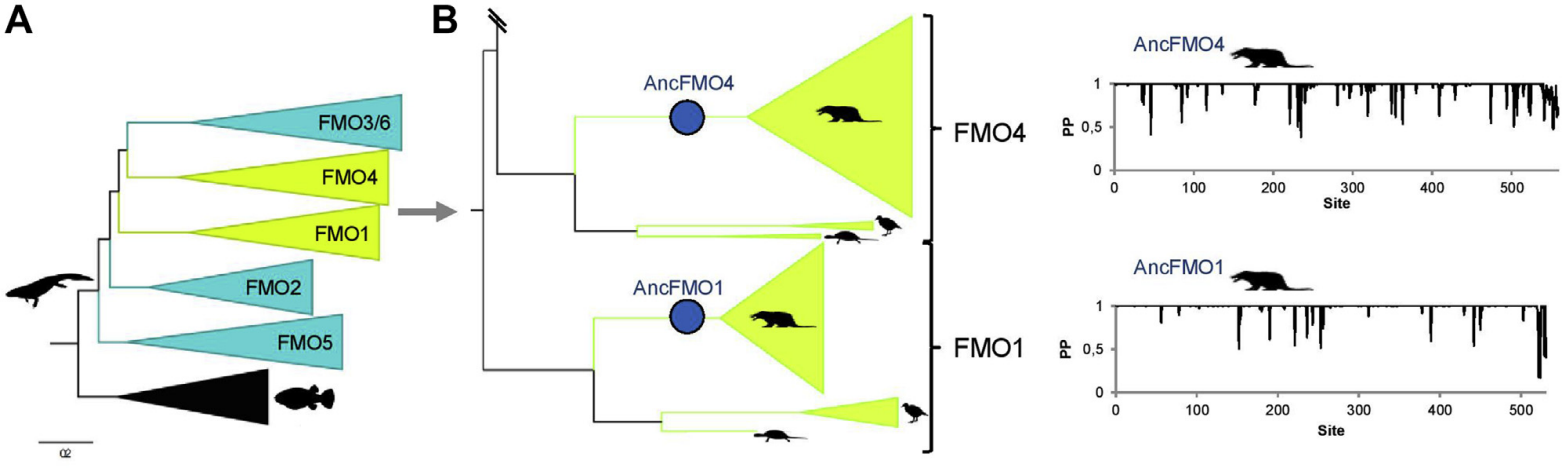
**Ancestral sequence reconstruction of mammalian FMOs**. *A*, Compressed tree of FMOs from jawed vertebrates. The tree depicts the explosion of FMOs in tetrapods. The five FMO clades are shown and those colored in green have been analyzed in this work. *B*, Close up of the FMO4 and FMO1 clades. Taxonomic distribution is depicted with silhouettes as follows: ancestral tetrapod (), bony fishes (), mammals (), aves () and testudines (). Reconstructed mammalian ancestors are shown with blue circles. On the right the corresponding graphs of the posterior probability distribution per site are shown.

Sequence analysis has shown that the FMO paralogs emerged at the time of tetrapod evolution and all five FMO paralogs were encoded in the genome of the mammalian ancestor, 177 mya (24) (Fig. 2, Fig. S1, Table S1). Ancestral flavin-containing monooxygenase (AncFMO)1 was reconstructed with high confidence (overall posterior probability [PP] of 0.95). This sequence shows the typical length exhibited by other mammalian FMOs (531 amino acids) and shares 89.8% sequence identity (54 residue changes) with hFMO1. We found that 10 sites were ambiguously reconstructed (Data S1). AncFMO4 was also reconstructed with high PP (0.97) with 24 ambiguously reconstructed sites (Data S1). This enzyme exhibits a C-terminal extension of approximately 20 amino acids, and its length was 559 amino acids. The sequence identity compared with hFMO4 was 89.8% (57 changes). Full-length AncFMO1 and AncFMO4 were selected for experimental characterization. Synthetic genes encoding proteins with the second-best state at each of the ambiguously reconstructed sites (alternative amino acids) were also obtained to produce the alternative versions (AltAncFMOs) and assess the robustness of the reconstruction. No expression of AncFMO4 or AltAncFMO4 was observed after growth at 24 °C or 17 °C. Lack of expression was confirmed through Western blot analysis by using a 6xHis-tag–fused peroxidase monoclonal primary antibody. The cell lysate and insoluble membrane pellets incubated with a range of detergents failed to show protein expression (Fig. S2). The characteristic C-terminal extension after the transmembrane domain apparently prevents its proper expression in agreement with previous literature (25). By contrast, up to 20 mg per liter of culture of AncFMO1 and AltAncFMO1 could be purified as FAD-containing proteins as evidenced by its intense yellow color. Furthermore, through Western blot analysis, AncFMO1 was demonstrated to be extracted from the membrane fraction using a range of different detergents (Fig. S2). The melting temperature (T_m_) of AncFMO1 was evaluated using a Tycho NT.6 system to assess its stability (see Experimental procedures). The AncFMOs that we studied previously displayed high thermal robustness (T_m_ of the native enzyme ranged between 53 and 60 °C). AncFMO1 instead exhibited a relatively low T_m_ of 47 °C that increased to 52 °C in the presence of 200-μM NADP^+^ (Table S2).

### Catalytic properties

We first verified whether AncFMO1 was functional by testing known substrates of hFMOs. Gratifyingly, we found that AncFMO1 is indeed active on benzydamine, an anti-inflammatory drug (26), exhibiting typical Michaelis–Menten kinetic behavior (Table 1, Fig. S3). A *k*_cat_ of 0.18 s^−1^ was obtained which is very similar to what was previously determined for FMO1 from the cynomolgus macaque (*Macaca fascicularis*) (0.17 s^−1^) (27). AncFMO1 was also found to accept the prototypical FMO substrate, thioanisole (0.19 s^−1^). The alternative FMO1 ancestor, AltAncFMO1, displayed a similar activity toward benzydamine and thioanisole, with a rate of 0.20 s^−1^ and 0.16 s^−1^ in presence of 0.4-mM substrate, respectively, thus demonstrating the robustness of the reconstruction. The nicotinamide cofactor specificity of AncFMO1 was also explored. The kinetic analysis revealed that both the nicotinamide cofactors, NADH and NADPH, are accepted as electron donors. Although similar rates of catalysis were obtained, the K_M_ for NADPH was significantly lower than the K_M_ for NADH (Table 1). In the absence of any organic substrate, AncFMO1 displayed a similar uncoupling (*i.e.*, the oxidation of NAD(P)H leading to the release of hydrogen peroxide) rate regardless of the cofactor, 0.13 and 0.11 s^−1^ for NADPH and NADH, respectively. Once again, the K_M_ for NADH was higher than that for NADPH (Table 1). It should be noted that hFMO1 was also previously shown to be highly uncoupled, producing H_2_O_2_ in higher amounts than hFMO2 and hFMO3 (28).

**Table 1:** Steady-state kinetic parameters for AncFMO1

Substrate	Fixed substrate	*k*_cat_ (s^−1^)	K_M_ (μM)	*k*_cat_/K_M_ (s^−1^/M)
aBenzydamine	0.10-mM NADPH	0.18 (0.004)	<13	>14,000
aThioanisole	0.10-mM NADPH	0.19 (0.007)	<25	>7600
bNADPH	1.0-mM thioanisole	0.26 (0.009)	<9	>29,000
bNADH	1.0-mM thioanisole	0.53 (0.03)	54 (8)	10,000
cNADPH	-	0.13 (0.01)	55 (13)	2400
cNADH	-	0.11 (0.009)	91 (17)	1200

AncFMO, ancestral flavin-containing monooxygenase.

a Reactions were followed by measuring the NADPH consumption at 340 nm and rates calculated using NADPH extinction coefficient 6.22 mM^−1^ cm^−1^.

b NAD(P)H steady-state kinetics in the presence of 1.0-mM thioanisole, rates followed at 340 nm.

c AncFMO1 uncoupling reaction with NAD(P)H, in the absence of the substrate. The values in brackets refer to SDs.

Having established that AncFMO1 is enzymatically active, we sought to further characterize the enzymatic conversions and the resulting products (Table S3). Benzydamine is converted to didesmethyl-benzydamine by CYPs, whereas hFMO1 transforms it into the corresponding N-oxide (26, 29). Using HPLC analysis, we found that also AncFMO1 converts benzydamine into its N-oxide. Thioanisole was converted into the corresponding sulfoxide with high enantioselectivity, producing mainly the (*R*)-enantiomer. To probe the activity of AncFMO1 toward a bulky molecule, tamoxifen (an antiestrogen drug (30)), was tested as a substrate. We found that tamoxifen was converted into the N-oxide derivative, as previously reported for hFMO1 (31). To test whether in addition to N- and S-oxidations AncFMO1 catalyzes also Baeyer–Villiger oxidations, two ketones were assessed as potential substrates, hepta-2-one and bicyclo[3.2.0]hept-2-en-6-one (32, 33). Although no conversion of hepta-2-one was observed, bicyclo[3.2.0]hept-2-en-6-one was partly converted, implying some moderate Baeyer–Villiger oxidation activity. Upon harvesting the cells, it became clear that a blue pigment had been formed as evidenced by colored cell pellets upon centrifugation. For microbial FMOs, it has been shown that they form indigo blue when expressed in *Escherichia coli* (34). Thus, it is most likely that AncFMO1 converts indole into indoxyl that spontaneously dimerizes to form indigo blue. To verify this, the purified enzyme was incubated for 1 h at 30 °C with indole and a NADPH regeneration system, which indeed resulted in formation of indigo (Fig. S4). Collectively, these data demonstrated that AncFMO1 featured all the typical properties of FMOs and was able to convert known FMO1 substrates.

### Rapid kinetics

**Figure 3:**
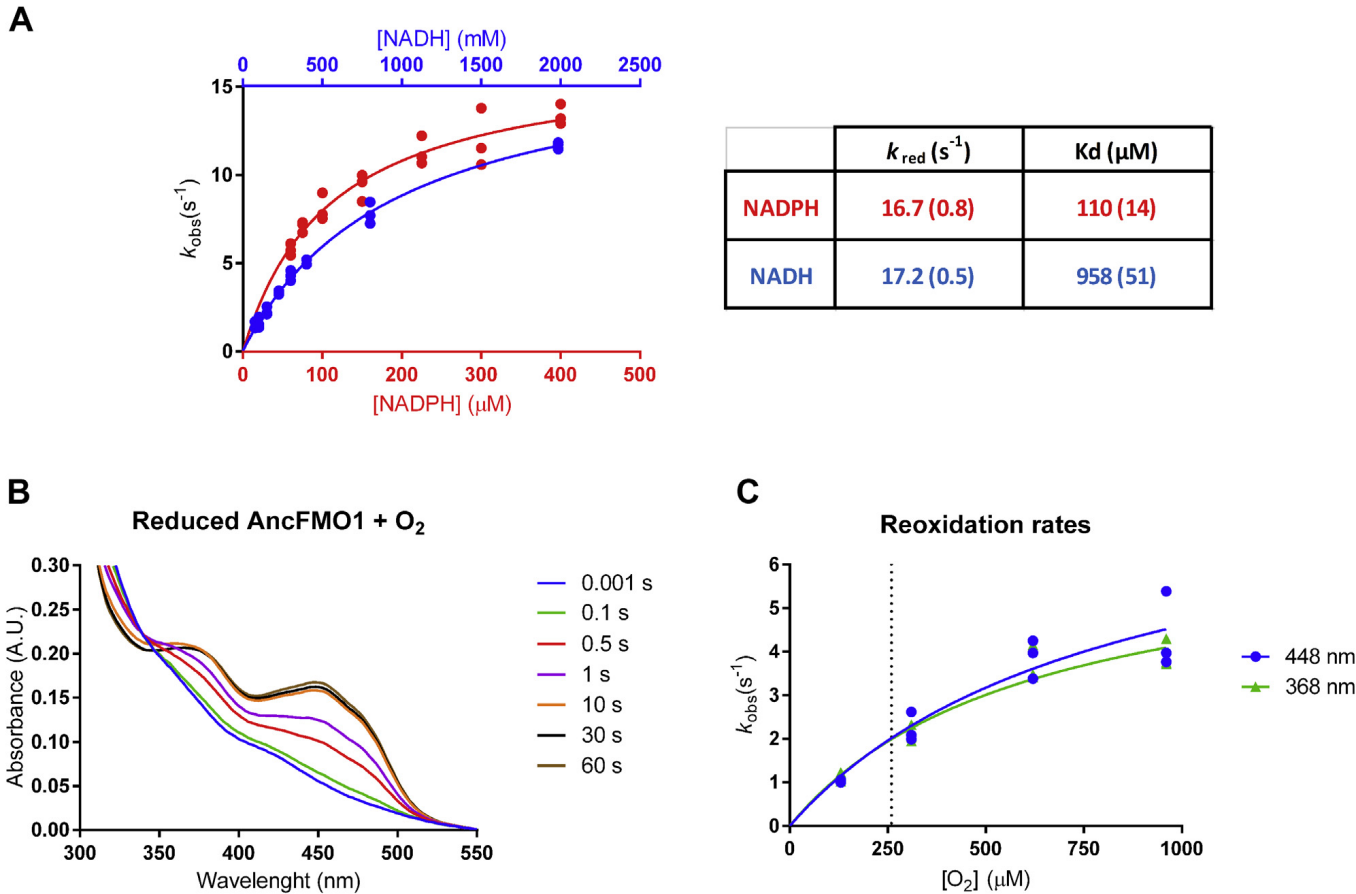
**AncFMO1 stopped-flow kinetics.***A*, reductive half-reaction rates measured in technical triplicates under anaerobic conditions with increasing concentrations of NADH (*blue points*) or NADPH (*red points*). The binding constants K_d_ and *k*_red_ values were calculated by fitting the reductive half reaction to the Michaelis–Menten equation with the SD reported in *brackets*. *B*, reoxidation spectra of the reduced AncFMO1 with oxygenated buffer over time. *C*, reoxidation rates with increasing concentration of dioxygen, observed at 368 nm (*green triangles*) and 448 nm (*blue points*) in technical triplicates. The *dotted lines* correspond to the atmospheric concentration of dioxygen (260 μM), present during steady-state kinetics. AncFMO, ancestral flavin-containing monooxygenase; FMO, flavin-containing monooxygenase.

Both the reductive and oxidative half reactions were investigated separately using the stopped-flow technique. The first steps of the catalytic cycle of FMOs involve the binding of NADPH after which the FAD is reduced through a direct hydride transfer. The reductive half reaction can be monitored spectrophotometrically by mixing the enzyme with NADPH under anoxic conditions because the reduction of the flavin can be easily measured by following the decrease of absorbance at 448 nm. We determined the reduction rates at different NADPH concentrations (Fig. 3*A*). Fitting the data resulted in a relatively fast reduction rate constant with *k*_red_ = 16.7 s^−1^ and a dissociation constant of 110 μM. The kinetic and spectral data indicate that flavin reduction takes place in a single irreversible hydride transfer step. We also monitored the reductive half reaction using NADH, resulting in a similar reduction rate constant (*k*_red_ = 17.2 s^−1^) and a dissociation constant of 958 μM, which is almost 9 times higher than when using NADPH. The presence of 1.0-mM thioanisole did not affect the rate of reduction, suggesting that flavin reduction precedes binding of substrate as has been proposed for other class B flavoprotein monooxygenases (13).

By preparing anaerobically reduced AncFMO1, the oxidative half reaction could be monitored by mixing it with dioxygen-containing buffer. For several FMOs, the reaction of reduced enzyme with dioxygen results in the formation of a C(4α)-hydroperoxyflavin intermediate with absorbance features around 360 to 380 nm (35). The intermediate subsequently decays at varying rates, depending on its stabilization by the enzyme. For AncFMO1, when mixed as reduced enzyme with dioxygen, a rapid increase of absorbance at both 368 nm and 448 nm was observed and we were unable to distinguish intermediate formation from the full reoxidation of the flavin (Fig. 3*B*). This shows that there was only a minor buildup of the C(4α)-hydroperoxyflavin intermediate and indicates that AncFMO1 is not good at stabilizing the oxygenating enzyme intermediate. This observation is consistent with the sustained uncoupling featured by AncFMO1 (Table 1). The rate of reoxidation of the reduced AncFMO1 in presence of 260-μM dioxygen was around 2 s^−1^ (Fig. 3*C*), whereas the steady-state kinetics of uncoupling suggested a slower reaction with a rate only reaching 0.12 s^−1^ (Table 1). These kinetic data show that AncFMO1 displays a similar kinetic behavior when compared with other FMOs.

### Overall three-dimensional structure of FMO1

**Figure 4:**
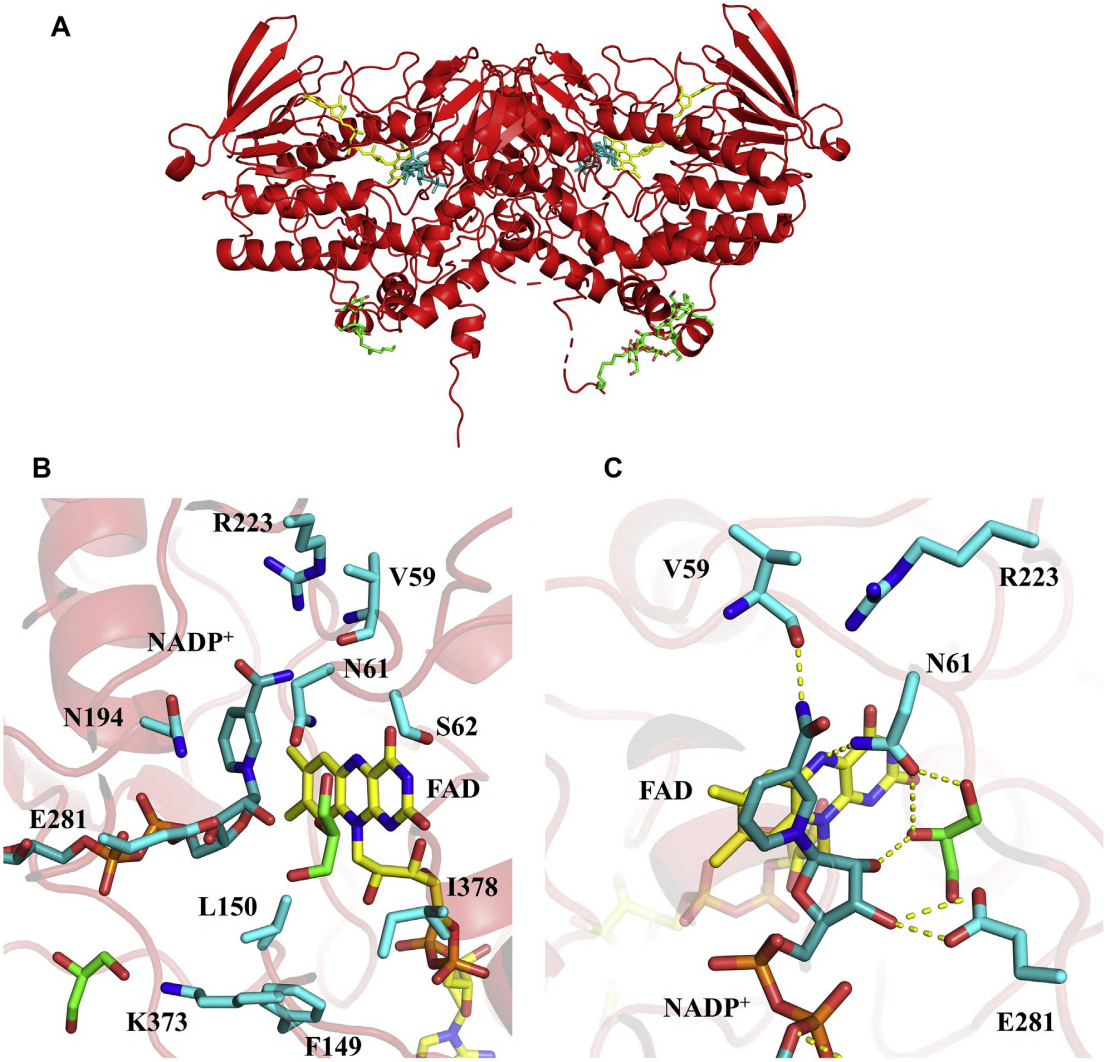
**Crystal structure of AncFMO1 and its active site.***A*, dimeric AncFMO1 with its partially mapped C-terminal helices pointing downward toward the membrane. *B*, the active site of AncFMO1 in the presence of oxidized coenzyme, NADP^+^, and two glycerol molecules (*green*) is depicted, with the key residues labeled. *C*, extensive hydrogen bond interactions between E281, N61, V59, FAD, NADP^+^, and a glycerol molecule are shown to illustrate key intermolecular interactions and potential substrate binding modes, represented by *dashed yellow lines*. FAD, NADP^+^, and DDM molecules are colored in *yellow*, *cornflower blue*, and *white*, respectively. AncFMO, ancestral flavin-containing monooxygenase; DDM, dodecyl-β-D-maltoside; FAD, flavin adenine dinucleotide.

We were able to successfully crystallize AncFMO1 and to solve its structure at 3.0 Å resolution (Fig. 4*A*, Table 2, Fig. S5). STARANISO, an anisotropy correction server, was critical to correct for the anisotropy of crystals leading to drastically improved electron density maps. The asymmetric unit consisted of two dimers (Table 2), and the dimerization observed for AncFMO1 is the same as for the dimers documented by the other AncFMO crystal structures (23) (Fig. 4*A*). Consistently, the structure of AncFMO1 possesses key structural domains and folds canonical to the mammalian FMOs (Fig. 4*A*). Alongside the well-conserved NAD(P)H- and FAD-binding domains, known as the paired Rossmann fold, AncFMO1 includes a large 80-residue insertion, two small α-helical triads that form the hydrophobic ridge that embeds into the phospholipid bilayer, and a large transmembrane C-terminal helix that drills into the membrane. In addition, in accordance with the previously described AncFMOs, AncFMO1 displayed large hydrophobic strips on the surface of the crystal structure that promote monotopic membrane association (Fig. S6) (22). The oxidized coenzyme, NADP^+^, was successfully bound in the crystal structure and two glycerol molecules were found located near/at the active site. Owing to weak electron density and a highly disordered peptide backbone, the C-terminal helices were poorly constructed in the crystal structure. Nevertheless, resolved detergent molecules demarcated the protein–membrane interface that was illustrated by other AncFMOs (23). In addition, pairwise structural superpositions between AncFMO1 and AncFMO3-6 monomers (sequence identity 59%) portrayed an RMSD of 0.8 A over 449 Cα atoms, corroborating a high protein scaffold similarity. The crystal structure revealed that the ambiguous sites of the AltAncFMO1 sequence were all peripheral from the active site and not expected to impact enzymatic activity. This is supported by the enzyme activities observed for AltAncFMO1.

**Table 2:** Data collection and refinement statistics

Data	AncFMO1 (PDB: 7AL4)	
Number of crystals	2	
	Aimless	STARANISO
Data collection^a^		
Resolution range (Å)^b^	49.100–3.000 (3.041–3.000)	
Space group	P 2_1_	
Unit cell (Å), (°)	115.92 92.453,156.69	90 95.122 90
Total reflections	437,615 (22,096)	-
Unique reflections	66,237 (3899)	39,983 (567)
Multiplicity	6.6 (5.0)	-
Completeness (%)	99.7 (98.8)	60.2 (14.1)
Completeness ellipsoidal (%)	-	99.5 (94.8)
Mean I/simga (I)	3.4 (0.3)	5.5 (1.1)
R_merge_	0.245 (7.993)	-
CC_1/2_	0.994 (0.028)	-
Refinement		
R-work (%)	-	22.8
R-free (%)	-	26.4
Number of non–hydrogen atoms^c^	-	17,079
RMS (bonds) Å	-	0.004
RMS (angles) °	-	1.359
Ramachandran favored (%)	-	82.6
Ramachandran allowed (%)	-	11.2
Ramachandran outliers (%)	-	6.2
Average B-factor	-	82

AncFMO, ancestral flavin-containing monooxygenase.

The final data set was measured by combining two previously merged reflection files, derived from two crystals, which were then corrected for anisotropy using STARANISO (49). The refined data are derived from the anisotropic corrected data. Values in parentheses are for the highest resolution shell. The asymmetric unit contained 4 protein molecules for AncFMO1.

a The data set was measured after merging reflection files from two crystals.

b Values in parentheses are for highest resolution shell.

c The asymmetric unit contained four protein molecules, respectively.

### Distinct features of the FMO1 active site

To gain insight into the degree of structural conservation among the mammalian FMOs, we inspected the active site of AncFMO1. First, the underlying architecture that encases the isoalloxazine ring of the FAD and anchors the nicotinamide ring of NAD(P)H is identical to the AncFMOs (Fig. 4, *B*–*C*). Furthermore, the active-site residues are well conserved among the FMOs and occupy similar conformations. Moreover, apart from Ile378 that is a methionine in hFMO1 (Fig. S7), all active site residues are conserved between AncFMO1 and hFMO1, making AncFMO1 a reliable model for the human counterpart.

Despite the likeness AncFMO1 shares with the other AncFMOs, the three-dimensional structure reveals some clear differences that render this enzyme somewhat distinct from its paralogs. Residues 149 and 150 that sit close to the ribityl tail of the isoalloxazine ring are distinct from other FMOs (Fig. 4, *B*–*C*). The previously described AncFMO crystal structures possessed two conserved histidine residues at these positions. His150 was shown to form a hydrogen bond with the highly conserved Gln373, which was observed to be also within hydrogen bond distance to the 3’-OH group of NADP^+^. Together, these two hydrogen bonds formed a key steric blockade that maintained the closed cavity feature that was a characteristic of mammalian FMOs (23). AncFMO1 possesses a phenylalanine and a leucine at positions 149 and 150, respectively, creating a hydrophobic and wider pocket at the base of the active site. Furthermore, with Leu150 not being able to create hydrogen bonds, Lys373 is not tied down and can extend outward, toward a glycerol molecule situated below the diphosphate moiety of the NADP^+^, thereby further enlarging the active site (Fig. 4*B*). With these residues being conserved between AncFMO1 and hFMO1, it is likely that these features would also be observed for hFMO1, therefore further validating the use of AncFMO1 as a structural model.

**Figure 5:**
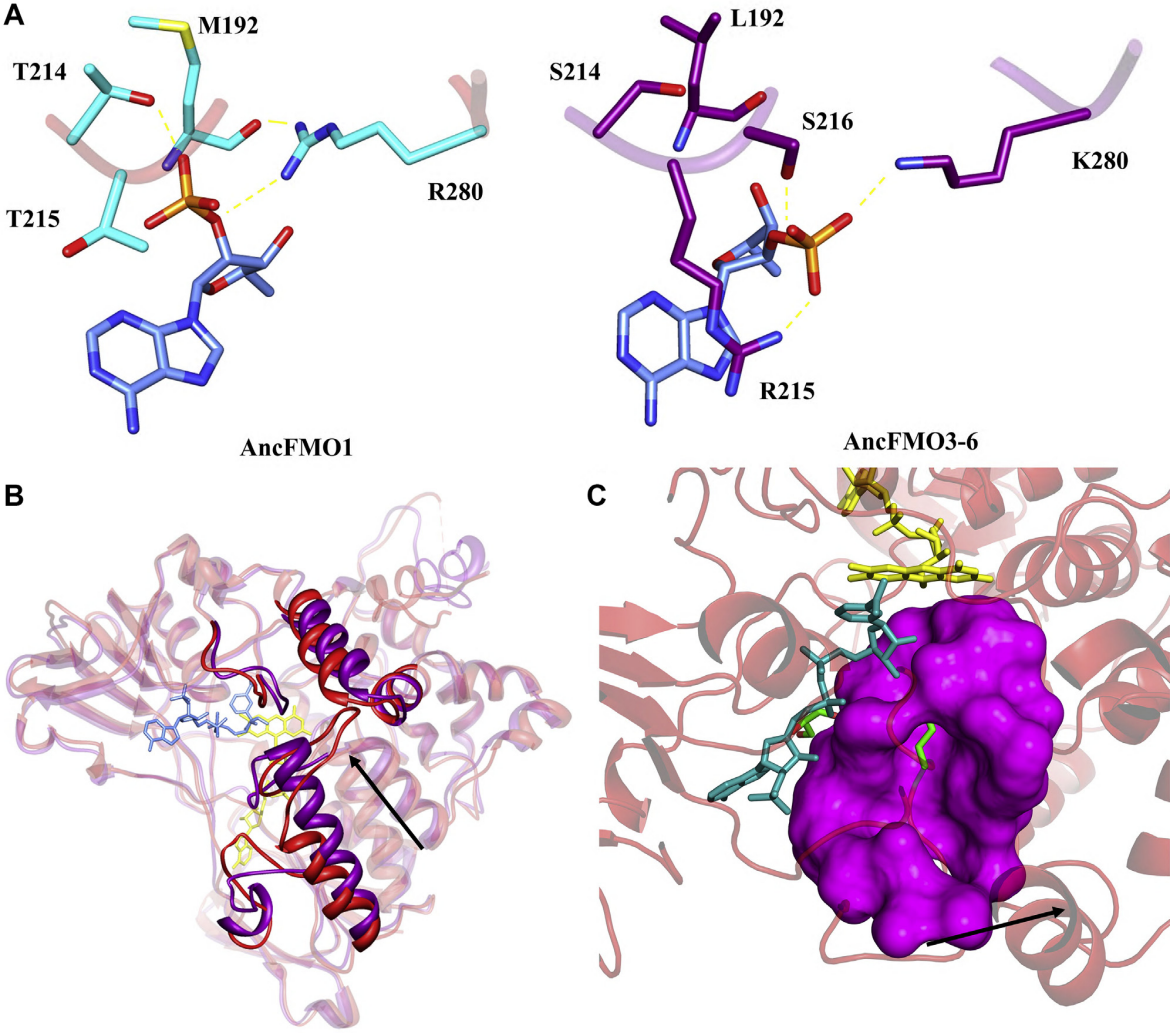
**Unique structural features of AncFMO1.***A*, the differing 2’-phosphate binding site of NADP^+^ for AncFMO1 (*left*, *side chains in cyan*) compared with AncFMO3-6 (PDB:6SE3) (*right*, *side chains in dark purple*) is shown with key hydrogen-bonding interactions shown as *yellow dashed lines*. NADP^+^ is shown in *cornflower blue*. *B*, the conformation adopted by residues 416 to 425 that reaches out toward the α-helical triad in a large arched conformation is indicated by the *black arrow*. Super positioning AncFMO1 (*red*) against AncFMO3-6 (*dark purple*) conveys the new structural topology. *C*, the large active site cavity (approximately 19 Å wide) is depicted in *magenta* and stretches out toward the solvent and the membrane–protein interface (indicated with a *black arrow*). The side chain of E281 is shown in *green* pointing toward the isoalloxazine ring and the active site. FAD, NADP^+^, and glycerol molecules are shown in *yellow*, *cornflower blue*, and *green*, respectively. AncFMO, ancestral flavin-containing monooxygenase; FAD, flavin adenine dinucleotide.

Commensurately with AncFMO1, hFMO1 has been previously described to use both NADPH and NADH as reducing coenzymes for oxidative catalysis (36). Intriguingly, the 2’-phosphate occupies a new conformation unique to AncFMO1 (Fig. 5*A*). While the other AncFMOs crystal structures portray the 2’-phosphate to be extended out into the solvent, in the AncFMO1 crystal structure, the 2’-phosphate is tucked inside the NADP^+^ binding domain. Typically, a highly conserved arginine residue (site 215 for AncFMO2 and AncFMO3-6) is observed to be within the vicinity of the 2’-phosphate binding pocket. A range of intermolecular interactions including hydrogen bonds and ionic interactions are then established between the guanidinium headgroup of the arginine side chain and the 2’-phosphate of the NADP^+^ molecule (23). AncFMO1, however, possesses a threonine at position 215 and a glycine at 216 (His, Ser, and Arg for AncFMO2, 3–6, and 5, respectively), which are unable to produce these key noncovalent interactions, resulting in the 2’-phosphate rotating upward to establish contacts. More specifically, the 2’-phosphate establishes hydrogen bonds with the side chains of Arg280 and Thr214, and the peptide of Met192 (Fig. 5*A*). Apart from Arg280 that is a chemically equivalent lysine in hFMO1, these residues are conserved between AncFMO1 and hFMO1; therefore, it is likely that the coenzyme would bind in the same conformation. The loss of key residues in the 2’-phosphate binding pocket likely contributes toward the abolishment of the coenzyme selectivity.

### AncFMO1 contains a porous and exposed active site

FMO1 has been described in literature to be the least selective of the FMO family, metabolizing a range of substrates such as tamoxifen, benzydamine, trifluoperazine, and sulindac sulfide (37), and unlike FMO2, is not limited or restricted to the substrate size or length (38). This finding substantiates that the active site of FMO1 is more susceptible and vulnerable to substrate exposure. To corroborate this speculation, we inspected the tunnels and cavities of AncFMO1 using HOLLOW, a program that facilitates the internal and external surface imaging of proteins (39). AncFMO1 exhibits a narrow tunnel that passes through the α-helical triad toward the membrane, corroborating that this FMO has the capacity to extract substrates from the membrane in a manner consistent with its paralogs. Nonetheless, unlike the other characterized crystal structures that conveyed closed active-site cavities, AncFMO1 exhibits a significantly porous active site. First of all, the above-described loss of a key hydrogen bond between residues 150 and 373 results in a *leaky* active site (Fig. 5, *B*–*C*). Moreover, the structural topology choreographed by residues 416 to 425 provides additional space for soluble substrates (Fig. 5*B*). Two hydrophobic residues, Phe420 and Leu422, protrude upward either side of the alpha helical triad and are anchored in place through a hydrogen bond interaction between the peptide backbone and the side chain of Thr249 (Fig. S8). As a result, this new fold creates a steric blockade underneath the α-helical triad, greatly restricting the ease of substrate access from the membrane. Despite the motif blocking a hydrophobic access point underneath the alpha helical triad, it could be conceived that the vicinity of this newly formed cavity to the protein–membrane interface (and the flexibility of the loop) may be able to siphon substrates dwelling in the membrane (Fig. 4, *B*–*C*). Moreover, this new conformation also greatly opens up the protein surface to the solvent and its flexibility and proximity to the protein–membrane interface may provide new entry points for substrate transit.

## Discussion

The first mammals on earth were armed with the arsenal of five FMOs to deal with harmful chemicals generated endogenously or from the natural environment. This group of detoxifying enzymes has been maintained across evolution in all species in the order, including humans. In our previous work, we characterized in depth the ancestors of hFMO2, hFMO3, and hFMO5. However, FMO1 and FMO4 remained uncharacterized until now. In this study, we performed ASR of FMO1 and FMO4. Although we could not express the ancestral form of FMO4, the AncFMO1 was successfully overexpressed and could be purified as stable and functional enzyme. This allowed us to study, for the first time, structural details of this mammalian FMO and correlate the structural features with its catalytic properties. Mammalian FMO1s have been shown to metabolize a wide variety of compounds, including various drug molecules. The conversion and steady-state kinetics conducted with benzydamine, tamoxifen, and thioanisole revealed that AncFMO1 accepts the same substrates as other mammalian FMOs with a similar rate and affinity (27, 36, 40). We therefore confirmed that the reconstructed enzyme is not troublesome to work with and can be used as a proper hFMO1 mimic with 89.5% sequence identity and almost full conservation of active site residues. Overall, the high sequence identity and the similarity in enzymatic catalysis between AncFMO1 and hFMO1 validate the use of AncFMO1 as a trustworthy structural surrogate for enzymatic assays and structure-based drug design. Also, this is the first multiaspect characterization of a mammalian FMO1.

The active site of AncFMO1 consists of key residues required for intermediate stabilization and coenzyme binding, including Arg223, Asn194, and Asn61. Serendipitously, the presence of a glycerol molecule in the active site highlights key hydrogen-bond interactions that could form during catalysis between the generated C(4α)-hydroperoxy flavin intermediate and a theoretical substrate. The distal hydroperoxy oxygen atom would engage with the oncoming soft nucleophile’s sp^3^-hybridized orbital, here represented as the central hydroxyl group of the glycerol molecule (Fig. 4, *B*–*C*). In addition, the 2’-OH group of the nicotinamide ribose of NADP^+^, that was speculated (41, 42) to stabilize the oxygenating intermediate, establishes a hydrogen bond with the same central hydroxy group of the glycerol. It could be assumed that the 2’-OH group of the nicotinamide ribose provides additional ligand binding roles such as orientating substrates for catalysis and positioning the nucleophilic center of the substrate for oxidation. The expanded volume of the inner chamber of the active site of FMO1 appears to be relatively inefficient at C(4α)-hydroperoxy flavin stabilization, thereby suffering from relatively high uncoupling.

Structural inspection of the NADP^+^ binding site inferred that AncFMO1 lacks multiple residues that have been illustrated in multiple nucleotide-binding enzymes to form contacts with the 2’-phosphate of NADP^+^. This deprivation promotes a conformational change with the 2’-phosphate relocating inward toward the NAD(P)H binding domain. Extensive work carried out by Dean and co-workers (43), on the evolutionary adaptation of isocitrate dehydrogenases for NAD^+^ over NADP^+^, demonstrated that removing a similar overhanging arginine residue that hydrogen bonds with the 2’-phosphate contributed toward NADH preference. Remarkably, despite AncFMO1 losing multiple key phosphate-binding partners in the NAD(P)H-binding domain, it is still able to utilize NADPH as a coenzyme. This fact clearly portrays that the 2’-phosphate contacts are not universal. In other words, the structural determinants of the cofactor acceptance are intrinsic to the nature of each enzyme family. During evolution, the FMO2, FMO3, and FMO5 paralogs developed, or retained, an exclusive specificity for NADPH. FMO1 might instead have developed a less-stringent specificity, or perhaps retained an ancestral dual cofactor usage. This leaves room for speculation with the differential expression of FMO1 at developmental stages in humans (44). Its ability to use both nicotinamide cofactors renders FMO1 a potent detoxifying enzyme by being able to use both pools of reducing cofactors.

Similarly to multiple aqueous xenobiotic degrading enzymes, AncFMO1 displayed a large solvent-accessible cavity with multiple entry points. The generation of this chamber is due to two structural features. First, the residue changes at the base of the active site (Leu150, Phe149, and Lys373,) create a hole in the vestibule and a *leaky* cavity. This is in turn emphasized by a large loop (residues 416–425) that further expands the substrate entry point. Collectively, these adaptations promote the transit of soluble compounds, suggesting FMO1 may have evolved to show a greater selectivity toward aqueous substrates. These elements and the crystal structure provide rationale behind the breadth of substrates documented for this FMO paralog, both hydrophobic and hydrophilic, and accentuate its xenobiotic detoxifying prowess.

Now that the structures of the ancestral forms of mammalian FMO1, FMO2, FMO3, and FMO5 have been elucidated, it becomes clear that these monooxygenases have similar catalytic-site architectures. Only the AncFMO5 structure revealed somewhat different features that may explain that FMO5 is an outlier concerning its catalytic properties, being able to efficiently catalyze Baeyer–Villiger oxidations. The high similarity of the inner part of the active-site cavity in AncFMO1, AncFMO2, and AncFMO3 coincides with an overlapping substrate specificity and reactivity (S- and N-oxidations). This seems to result in a set of redundant enzymes. Yet, inspection of their structures suggests that the outer segments of the active-site tunnels differ in their local topology, modulating accessibility and substrate preferences. This strategy has resulted in FMOs that can metabolize different classes of xenobiotics.

## Experimental procedures

### Ancestral-sequence reconstruction

The FMO phylogeny of jawed vertebrate previously reported was used as the starting point (23). The data set was constructed as follows: (i) previously experimentally characterized *Homo sapiens* FMO sequences were used as queries in BLASTp searches in GenBank nonredundant protein sequences (nr) and in UniProt KB. The searches were restricted by the taxonomy of organisms by classes or orders aiming to mine the whole diversity included in the terrestrial vertebrates (*i.e*., Amphibia, Aves, and Mammalia classes and Testudines order) guided by the TimeTree knowledge database (24). Specifically, the 26 orders of mammals were carefully scrutinized. Hits were collected when showing >55% identity (>80% coverage) and E-value=0.0. (ii) Partial sequences (<510 amino acid length) or poor-quality ones were excluded. (iii) All collected sequences were gathered and analyzed in a multiple sequence alignment using the MAFFT v7 software, removing those that were collected more than once. In the case of amphibian sequences, as more than 5 paralogs were detected, the genomic location and context were analyzed to rule out annotation artifacts. Those sequences corresponding to true ORFs were kept in the data set. This process allowed building a representative and nonredundant data set. The data set included 37 FMO1-like and 49 FMO4-like sequences from mammals. The multiple sequence alignment contained 365 sequences and 569 sites. Best-fit model parameters were obtained by the Akaike information criterion in ProtTest, v3.4. Phylogeny was inferred by the maximum likelihood method in RAxML v0.6.0 (1000 bootstraps) and subjected to transfer bootstrap expectation in BOOSTER. ASR was performed as marginal reconstruction using the maximum likelihood inference method in PAMLX, v.4.9. Sequences were analyzed using an empirical substitution matrix and empirical equilibrium amino acid frequencies (model = 3), 4 gamma categories, and LG substitution matrix. The PP distribution of ancestral states at each site was analyzed at nodes corresponding to mammalian AncFMO1 and AncFMO4. The length of the ancestors was treated by parsimony analyzing the presence/absence of gaps in the targeted nodes on the basis of the length of the derived sequences in each clade. This allowed us to determine that AncFMO4 had a C-terminal extension after the predicted transmembrane domain, which is a unique feature of mammalian FMO4s. Sites were considered ambiguously reconstructed when the alternative states displayed PPs > 0.2.

### Chemicals

All chemicals were ordered from Merck, whereas NEB10β cell strains and DNA ligase were from New England Biolabs. NADPH and NADP^+^ were ordered at Oriental Yeast Co.

### Cloning, transformation, and expression

Synthetic genes containing BsaI restriction sites at both the 5’ and 3’ ends were ordered from Integrated DNA Technologies. Genes were cloned following the Golden Gate cloning method. The recipient vector was a pBAD plasmid modified in such a way that the target protein is expressed fused at its N-terminus to a SUMO protein that carries a 6xHis-tag at its N-terminus. The cloning mixture was the following: 55.3 ng and 58.2 ng of AncFMO1 or AncFMO4 inserts, respectively, 75 ng of Golden Gate entry vector (a molar ratio of 2:1 insert:vector), 15 U BsaI-HF, 15 U T4 DNA ligase, T4 DNA ligase buffer (1×), and nuclease-free water added to a final volume of 20 μl. A negative control was prepared without any inserts, and the number of used cycles aimed for maximum efficiency: the first step with a cycle at 37 °C for 5 min was followed by 16 °C for 10 min, which was repeated 30 times. Then the temperature was set at 55 °C for 10 min and finally at 65 °C for 20 min. The sample was stored at 8 °C until the next day. Once cloned, the pBAD-6xHis-SUMO-AncFMO1/4 plasmids were transformed into NEB10β CaCl_2_ competent cells. 5.0 μl of plasmid DNA was added to 100-μl CaCl_2_ competent cells and incubated for 30 min. The cells were then heat shocked at 42 °C for 30 s and incubated on ice for 5 min. About 250-μl LB-SOC (45) was added to allow the cells to recover at 37 °C for 1 h. The resuspended cell pellets were then plated on LB-agar (45) containing 100 μg.ml^−1^ ampicillin and incubated overnight at 37 °C. Plasmids were isolated and verified by sequencing and a 20% glycerol stock was stored at −70 °C. A preinoculum of 4-ml LB-amp (50 μg.ml^−1^) was grown overnight at 37 °C and used to inoculate 2-L baffled flasks containing 400 ml of Terrific Broth medium (45), supplemented with 50 mg.l^−1^ ampicillin and incubated at 17 or 24 °C. Expression was induced by adding 0.02% L-arabinose from a sterile 20% stock (w/v) when the absorbance at 600 nm was between 0.2 and 0.5. Cultures were grown at 24 °C with shaking for a total of 30 h before harvesting. Incubating the cells at 17 °C significantly increased the yield of AncFMO1 and its detergent exchangeability, compared with those grown at 24 °C, later found to be crucial for crystallization. Cells were harvested by centrifugation (5,000*g*, 15 min, 10 °C), flash-frozen in liquid nitrogen, and stored at −20 °C.

### Cell disruption, membrane extraction, and purification

All the following steps were carried out on ice or at 4 °C. Cell pellets were resuspended into buffer A (250-mM NaCl, 50-mM potassium phosphate, pH 7.5) with a 5:1 ratio volume (ml): mass (g) and supplemented with 0.10-mM PMSF and 1.0 mM β-mercaptoethanol to prevent protein degradation. Cell disruption was performed by sonication (70% amplitude, 5 s ON, 5 s OFF, for a total of 20 min) or a high-pressure homogenizer (2 runs). After centrifuging at 18,000*g* for 20 min, the supernatant was removed and the pellet was resuspended into buffer A2 (250-mM NaCl, 50-mM potassium phosphate, 0.5% Triton X100 [TRX]-reduced, pH 7.5) with the same ratio as before (5:1). The resuspended pellet was mixed overnight at 4 °C to solubilize the membrane protein and centrifuged at 18,000*g* to collect the supernatant. AncFMO1 and AltAncFMO1 were purified with a metal-ion affinity chromatography that bound the histidine tag attached to the N-terminal part of the fused SUMO protein. The cell-free extract was applied to the column and washed with increasing concentrations of imidazole. Buffer B contained 250-mM NaCl, 50-mM potassium phosphate, 300-mM imidazole, and 0.5% TRX-reduced, pH 7.5. After the washing steps of 0-, 10-, and 50-mM imidazole, the protein was finally eluted with 300-mM imidazole. The elution buffer was exchanged with a storage buffer using a desalting column (250-mM NaCl, 50-mM potassium phosphate, 0.05% TRX-reduced, pH 7.5).

The purified 6xHis-SUMO tagged enzyme was frozen with liquid nitrogen and kept at −20 °C. Enzymatic assays were performed using these aliquots. Crystallization trials required further purification, including 6xHis-SUMO tag cleavage, and were carried out as outlined in our previous work (23). Purification of AncFMO1 was performed using dodecyl-β-D-maltoside (DDM) (0.03% w/v analytical grade). Exchanging the detergent from TRX to DDM was only successful for proteins that had been produced in cultures grown at 17 °C. Protein produced at 24 °C did not fully exchange TRX, and the heterogeneous solution was problematic during crystallization trials.

### Western blot analysis

Cells were lysed as described above with both the aqueous layer and membrane pellet kept for analysis. The insoluble membrane pellet was resuspended in buffer A to a final volume of approximately 60 ml. Resuspended membranes were aliquoted in eight 7-ml solutions and incubated individually overnight at 4 °C with the following detergents (1% (v/v) final) AncFMO4: SDS, DDM, TRX, octyl glucoside, FOS-Choline 8, glyco-diosgenin, lauryldimethylamine oxide, and dimethyldecyl phosphine oxide. AncFMO1: SDS, TRX, DDM, CYMAL-6, and octyl glucoside. The control fraction represents the membrane fraction without any added detergent. Solutions were pelleted to remove the insoluble components by spinning down the samples at 100,000*g* for 30 min at 4 °C. 20 μl from each sample (including the aqueous supernatant derived from the AncFMO4 expression test representing the aqueous fractions) were then submitted to SDS-PAGE analysis. The resulting gels were then transferred to a Mini Format, 0.2-μm PVDF, single-application (Bio-Rad) membrane using a Trans-Blot Turbo Pack and a Trans-Blot Turbo Transfer System (Bio-Rad). The membrane was then washed with a milky solution comprising milk powder (2.5% (w/v) Nestle), Tween-20 (0.05% (v/v)), and a Tris-buffered saline solution (50-mM Tris Cl, pH 7.5, 150-mM NaCl), final volume 50 ml, for 1 h. This step is important for preventing nonspecific antibody binding. The solution was then washed with the same solution including an anti–6xHis-tag–fused peroxidase monoclonal primary antibody (final volume 15 ml) for 1 h. The membrane was then washed with the same buffer excluding the antibody and milk powder to remove any excess milk and antibody unattached. The membrane was finally washed with the Clarity, Western Enhanced chemiluminescence substrate (Bio-Rad) to initiate chemiluminescence according to the manufacturer’s instructions to visualize the bands.

### Melting temperature assays

The T_m_ of AncFMO1 was assessed and determined using a Tycho NT.6 system (NanoTemper Technologies GmbH, Munich, Germany) in the absence and presence of 200-μM NADP^+^, respectively. Concentrations of AncFMO1 were determined using ε_FAD_ = 12.0 mM^−1^.cm^−1^ at 442 nm. Experiments were performed in triplicate, with each sample containing AncFMO1 (1.0 mg ml^−1^, determined using the calculated molecular weight of AncFMO1, 61 kDa), with or without NADP^+^ (200 μM), made to a final volume of 10 μl using the storage buffer. To ensure the T_m_ of AncFMO1 assessed using the Tycho NT.6 system was comparable to the ThermoFAD assay performed on the AncFMOs (23), a control experiment was performed using AncFMO2, which corroborated the previously observed T_m_ values (data not shown).

### Enzyme assays

All reaction components were prepared in the same buffer as the one used for enzyme storage: 250-mM NaCl, 50-mM potassium phosphate, 0.05% TRX-reduced, pH 7.5. Steady-state kinetics measurements were performed on a Jasco V-660 spectrophotometer in technical triplicates. Unless stated otherwise, AncFMO1 activity was measured by monitoring NADPH consumption at 25 °C (at 340 nm, ε_NADPH_ = 6.22 mM^−1^.cm^−1^). The reaction mixture comprised 0.10-mM NADPH (or NADH), 2.5- to 1000-μM substrate, and 0.01- to 0.1-μM enzyme. Catalytic activity of AltAncFMO1 was measured with the same settings as AncFMO1. The conditions were chosen according to literature to have a fair comparison with the extant hFMO. NAD(P)H uncoupling rates were obtained in absence of any substrate. K_M_ and *k*_cat_ values were calculated by fitting the data with the Michaelis-Menten equation using GraphPad 6.07 (La Jolla, CA). When it was not possible to measure further, the K_M_ was reported as below the lowest substrate concentration for which a rate could be determined.

### Conversions

Substrate conversions were performed at pH 7.5, using 5.0-mM substrate (1% MeOH), 0.10-mM NADPH, 2.0-μM enzyme, 5.0-μM phosphite dehydrogenase, and 20-mM sodium phosphite. The last two components were used as a regeneration system for NADPH, and the control did not contain any AncFMO1 protein. The final reaction volume was adjusted to 1.0 ml with the buffer and put into 4-ml vials before being incubated at 30 °C, with shaking, for 18 h. Conversion of thioanisole, heptan-2-one, and bicyclo[3.2.0]hept-2-en-6-one could be analyzed by GC-MS, whereas benzydamine and tamoxifen conversions were monitored by HPLC. To determine the enantioselectivity in the sulfoxidation of thioanisole, chiral HPLC analysis was performed. For GC-MS, compounds were extracted twice by adding one volume of ethyl acetate, vortexing for 20 s and centrifuging and eluting the organic phase through anhydrous magnesium sulfate. GC-MS analyses were performed using an HP-1 Agilent column (30 m × 0.25 mm × 0.25 μm). For thioanisole, the method was the following: injector and detector temperature at 250 °C, a split ratio of 5.0, and an injection volume of 1 μl. The column temperature was held at 50 °C for 4 min, increased by 10 °C/min to 250 °C and held for 5 min. Thioanisole and methyl phenyl sulfoxide had a retention time of 10.05 min and 13.60 min, respectively.

HPLC analyses were performed after diluting 100 μl of the sample into 400 μl acetonitrile, vortexing it for 20 s and centrifuging. Analysis was performed using reversed-phase HPLC. Samples were injected with a volume of 10 μl onto a JASCO HPLC system, equipped with a Grace Alltima HP C18 column (5 μm, 4.6 × 250 mm). The solvents used were water with 0.1% v/v formic acid (A) and acetonitrile (B), and the flow rate was 0.8 ml.min^−1^. For benzydamine, the method was the following: 45 min on a gradient of 25 to 95% B, 3 min with 95% B followed by a 3 min decreased gradient of 95 to 25% B, and finally a re-equilibration for 2 min. Benzydamine and benzydamine N-oxide were detected at 308 nm with retention time of 13.08 min and 13.82 min, respectively. For tamoxifen, the method was the following: 30 min on a gradient of 40 to 95% B, 3 min with 95% B followed by a 5 min decreased gradient of 95 to 40% B, and finally a re-equilibration for 2 min. Tamoxifen and tamoxifen N-oxide were detected at 276 nm with retention time of 13.06 min and 14.20 min, respectively.

Enantiomeric excess values were determined by chiral HPLC analysis using a Shimadzu LC-10ADVP HPLC equipped with a Chiralcel OD-H column (5 μm, 4.6 × 250 mm). For the chiral HPLC, the GC-MS sample was evaporated and resuspended into isopropanol. The method was heptane/isopropanol 90:10 with a flow rate of 1 ml.min^−1^. The *R*- and *S*- enantiomers of methyl phenyl sulfoxide were detected at 220 nm with retention time of 10.34 min and 13.04 min, respectively.

### Rapid kinetics

Stopped-flow experiments were carried out on a SX20 stopped-flow spectrophotometer equipped with a single-channel photomultiplier or a photodiode array detection module (Applied Photophysics, Surrey, UK). Solutions were prepared in 50-mM potassium phosphate, 250-mM NaCl, and 0.05% TRX-reduced, pH 7.5, and unless mentioned, the experimental design followed the protocol as published previously (23). Experiments were run with at 25 °C, with 5- to 15-μM enzyme, in technical triplicates. The *k*_red_ and K_d_ for AncFMO1 were determined by mixing anaerobically the enzyme with NAD(P)H and following the absorbance decrease at 448 nm. To probe the effect of substrate on the rate of reduction, the reduction of AncFMO1 by 100-μM NAD(P)H was followed both with and without 1.0-mM thioanisole. The oxidative half reaction was monitored using the photodiode array module that allows collecting absorbance spectra every millisecond. To prepare reduced AncFMO1 for the oxidative half reaction, dithionite was added to an anaerobic solution containing AncFMO1 and an equivalent amount of NADP^+^. Dithionite was titrated until the loss of the yellow color of the oxidized FAD was complete, indicating complete reduction to FADH_2_. Mixing of reduced AncFMO1 with dioxygen-containing buffers (130 μM dioxygen, final) was performed both with and without 100-μM NADP^+^.

### Crystallization and structural determination of AncFMO1

The crystallization condition that resulted in AncFMO1 crystals that displayed the highest diffraction is described below. Concentrations of protein were measured using the absorbance of the FAD at 448 nm, using an extinction coefficient of 12 mM^−1^ cm^−1^. Before crystallization, NADP^+^ (1.0 mM final) was incubated with AncFMO1 (12.5 mg ml^−1^, in storage buffer conditions containing 0.03% (w/v) DDM) for 1 h at 4 °C. 1 μl protein-containing solution was then mixed with 1 μl of the crystallization condition comprising 100-mM Hepes (pH 7.5), PEG 4000 (10% w/v), and glycerol (20% v/v) as cryoprotectant, in a sitting drop at 20 °C. The same crystallization solution was used as reservoir solution (1 ml). After 2 days, large yellow crystals formed. Crystals were then fished directly from the drop with no additional cryoprotectants.

Data were collected at the Swiss Light Source (Villigen, Switzerland) and processed with the XDS (46) and CCP4 packages (47). STARANISO was used as, similarly to AncFMO2, these crystals suffered severely from anisotropy (48, 49). Using STARANISO on the final merged data set was imperative for elucidating a good electron density map. The phase problem was solved by molecular replacement using AncFMO3-6 (PDB 6SE3) as a search model using Phaser (23, 50). The phases were greatly improved by density averaging with DM (47, 50). Model building and refinement were then conducted using COOT (51), Buccaneer (47), and Refmac5 (52). The residue outliers in the Ramachandran plots were 5.9% for AncFMO1. Figures were generated using UCSF Chimera (53), PyMOL (DeLano Scientific; www.pymol.org), and CCP4mg (47). Coordinates of the refined model of AncFMO1 were submitted to the protein data bank with PDB code 7AL4.

## Data availability

All data are contained within the manuscript.

## Conflict of interest

The authors declare that they have no conflicts of interest with the contents of this article.
