# Supplementary material for: Ancestral reconstruction of mammalian FMO1 enables structural determination, revealing unique features that explain its catalytic properties
Source: J Biol Chem. 2020 Dec 25;296:100221. doi: 10.1074/jbc.RA120.016297 (PMC7948450; doi:10.1074/jbc.RA120.016297)
Supplement: Supplementary Information [file mmc1.pdf]

Supplementary Information

Ancestral reconstruction of mammalian FMO1: unique structural features explain its catalytic properties

Gautier Bailleul[a], Callum R. Nicoll[b], María Laura Mascotti[a,c], Andrea Mattevi[b], Marco W. Fraaije[a,*]

[a] Molecular Enzymology group, University of Groningen, Nijenborgh 4, 9747AG, Groningen, The Netherlands
[b] Department of Biology and Biotechnology "Lazzaro Spallanzani", University of Pavia, Pavia, Italy
[c] IMIBIO-SL CONICET, Facultad de Química Bioquímica y Farmacia, Universidad Nacional de San Luis, San Luis, Argentina

Contents

Supplementary Information S1
Figure S1. Jawed vertebrates phylogeny of FMOs S2
Figure S2. AncFMO4 expression and solubilization screening of AncFMO1 ..... S4
Figure S3. Steady state kinetic plots for AncFMO1 ..... S5
Figure S4. Indigo formation. S6
Figure S5. Crystal structure of AncFMO1. ..... S7
Figure S6. Charged surface distribution for the crystallographic dimer of AncFMO1. ..... S8
Figure S7. Key residue changes between AncFMO1 and hFMO1. ..... S9
Figure S8. Overarching loop. S10
Figure S9. Gel filtration chromatogram of AncFMO1. ..... S11
Figure S10. Differing residues between AncFMO1 and hFMO1. ..... S12
Data S1. AncFMO sequences and lists of ambiguously reconstructed sites. S14
Table S1. Jawed vertebrates FMOs dataset. S22
Table S2. Melting temperatures of the AncFMOs. S23
Table S3. Conversions catalyzed by AncFMO1 ..... S24

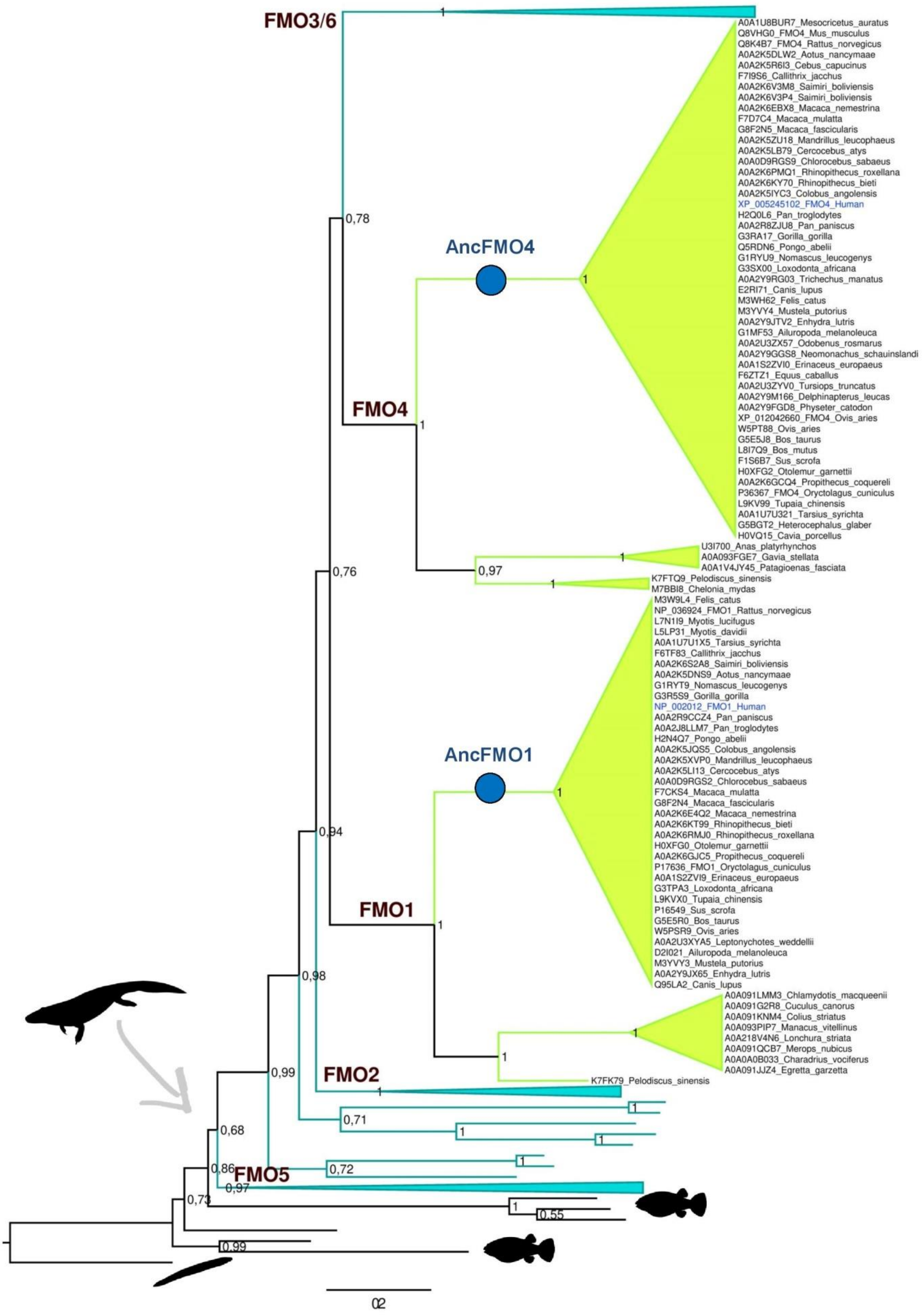

Figure S1. Jawed vertebrates phylogeny of FMOs. Tree was constructed in RAxML v0.6.0, 1000 bootstraps were run and subjected to TBE (shown at the nodes). The employed MSA was trimmed in single sequence extensions and contained 365 taxa and 569 sites. Clades are collapsed and colored

according to the paralog groups: FMO2, FMO3/6 and FMO5 (teal), FMO1 and FMO4 (lime). Uniprot accession codes and species names are provided for all sequences in FMO1 and FMO4 clades. The emergence of terrestrial vertebrates is depicted with an arrow and a silhouette (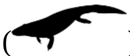). The tree was rooted according to the species tree in cephalochordates (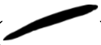). The tree was prepared in Figtree v1.4.2 and silhouettes obtained from http://www.phylopic.org/.

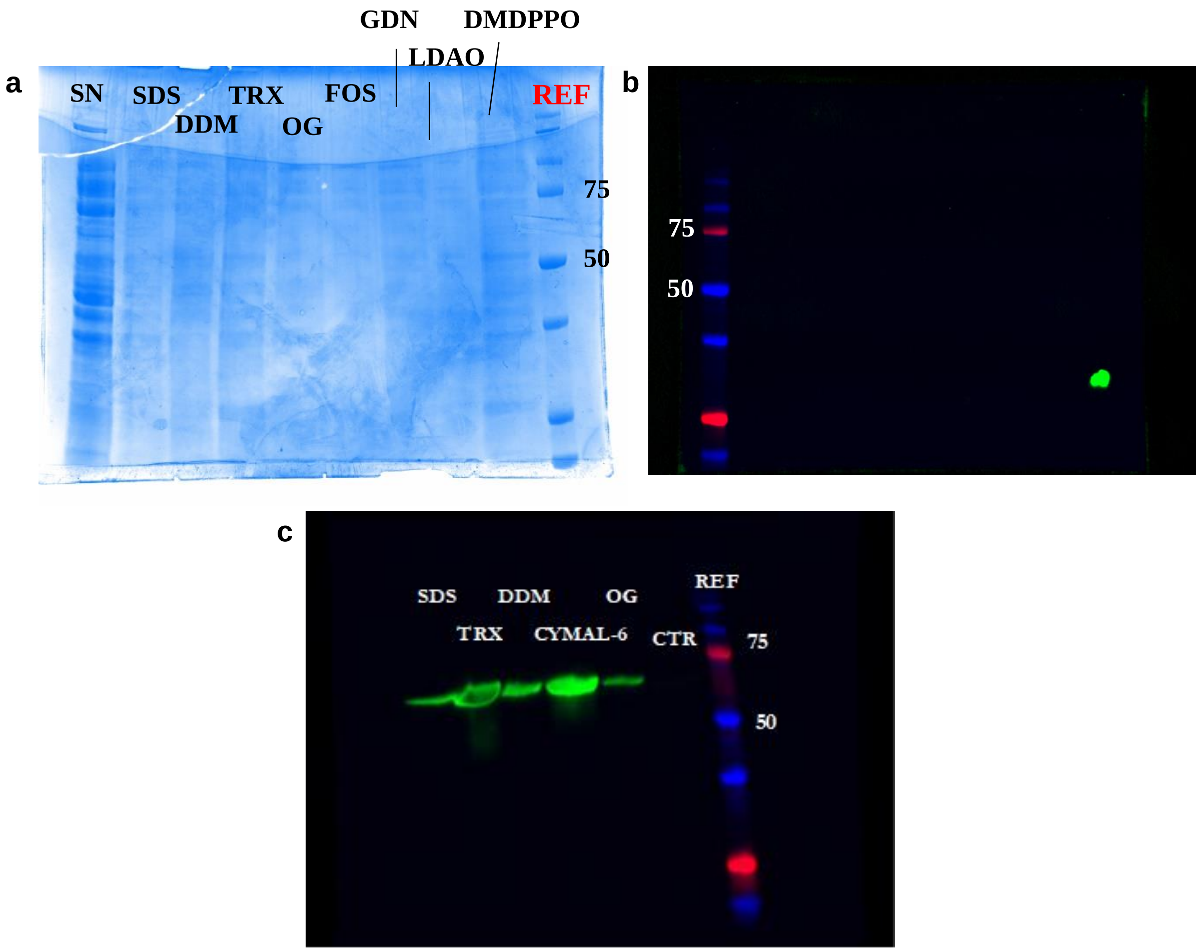

Figure S2. Analysis of AncFMO4 expression and solubilization screening of AncFMO1. **a**, SDS-PAGE corresponding to the various detergents used to solubilize AncFMO4 from the membranes, including the aqueous supernatant. **b**, Western-blot of the SDS-PAGE, with the image flipped with the lanes in reverse. Detergents used; SN (supernatant), SDS (sodium dodecyl sulfate), DDM (dodecyl-beta-maltoside), TRX (Triton-X 100), OG (octyl glucoside), FOS (FOS-Choline 8), GDN (glyco-diosgenin), LDAO (lauryldimethylamine oxide) and DMDPPO (dimethyldecyl phosphine oxide). For clarity, certain lanes are indicated with black lines. **c**, Western-blot detergent screening for AncFMO1 extraction. Native membranes collected during purification were resuspended and incubated overnight with various detergents including sodium dodecyl sulfate (SDS), Triton-X 100 (TRX), dodecyl beta-maltoside (DDM), CYMAL-6 and octyl glucoside (OG). CTR refers to the control sample without any detergent addition. REF refers to the standard molecular weight markers, with bands of 75 and 50 kDa indicated. 6xHis-tag antibodies were used to generate fluorescence.

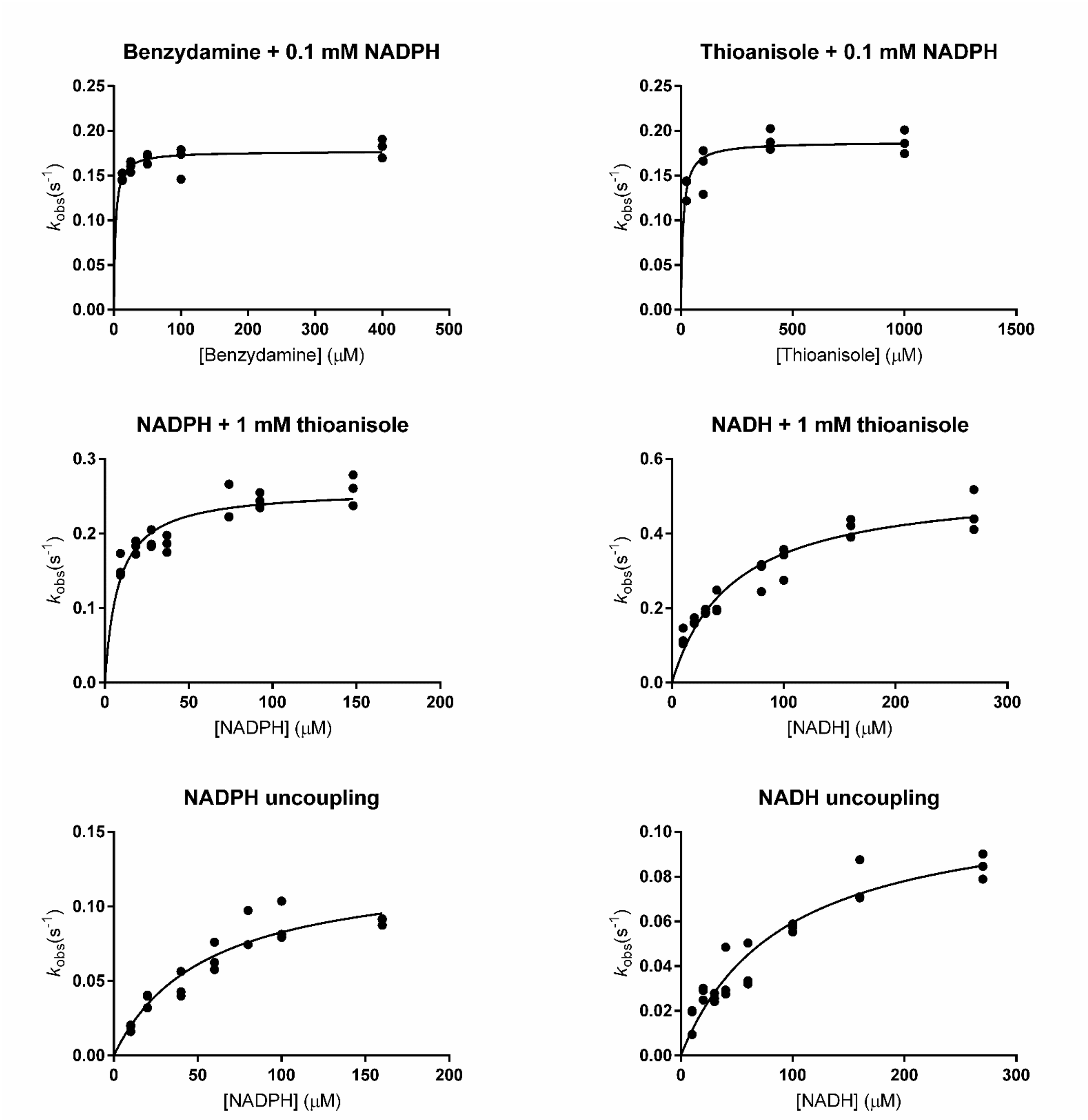

Figure S3. Steady state kinetic plots for AncFMO1. Reactions were followed by measuring the NAD(P)H consumption at 340 nm and rates calculated using NAD(P)H extinction coefficient 6.22 $mM^{-1}.cm^{-1}$. The plots were obtained by fitting the measured NAD(P)H depletion rates triplicates to GraphPad. Standard deviations are reported in Table 1.

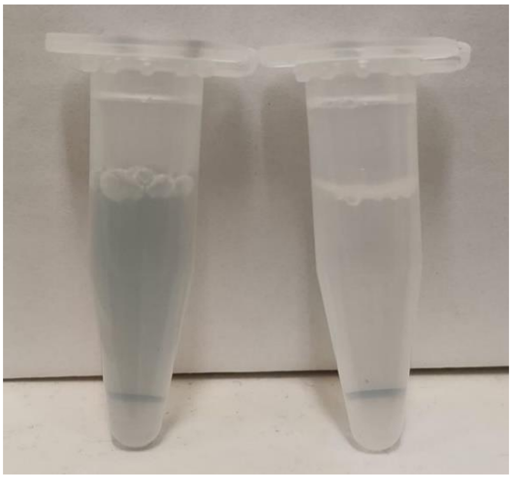

Figure S4. Indigo formation. The tube on the left contained 100 µM NADPH, 20 mM phosphite, 5 µM PTDH, 1 mM indole and 2 µM AncFMO1. The control tube on the right only lacks AncFMO1. The picture was taken 1 hour after addition of enzyme, at 30 ºC. All components were prepared in buffer (250 mM NaCl, 50 mM potassium phosphate, 0.05% Triton X100-reduced, pH 7.5).

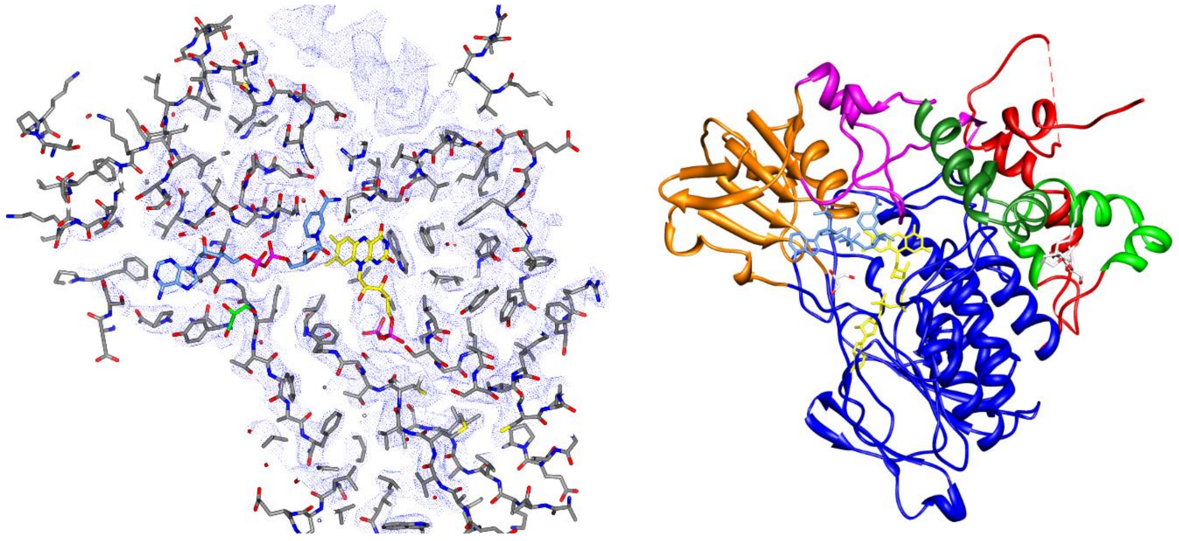

Figure S5. Crystal structure of AncFMO1. Left, Electron density maps of the AncFMOs. The depicted 2Fo-Fc maps (shown in blue) were calculated by averaging the electron density maps obtained after molecular replacement, and anisotropic correction using Staraniso. The crystal structure of AncFMO3-6 (PDB: 6SE3) was used as model for molecular replacement after the scaled reflections had been corrected for anisotropy using Staraniso. FAD and $NADP^+$ are shown in yellow and cornflower blue respectively. The contour level is 1.4 σ. The glycerol molecule shown and the amino acids are shown in green and grey, respectively. **Right**, AncFMO1 depicted as a monomer with its domains and subdomains colored. Blue and orange colors represent the well conserved FAD (residues 2–154 and 331–442) and NAD(P)H (residues 155–213 and 296–330) binding domains, respectively. The subdomains colored in pink and dark green portray the distinct 80-residue insertion (residues 214-295) associated to mammalian FMOs. The dark green subdomain (residues 231-261) represents the hydrophobic substrate access points. Together, the light green (residues 449-473) and dark green subdomains form a large hydrophobic strip that grapples onto the membrane. Glycerol and DDM molecules are shown in white. Finally, the red-colored subdomain (residues 474-532) represents the C-terminus. FAD and $NADP^+$ are colored in yellow and cornflower blue, respectively.

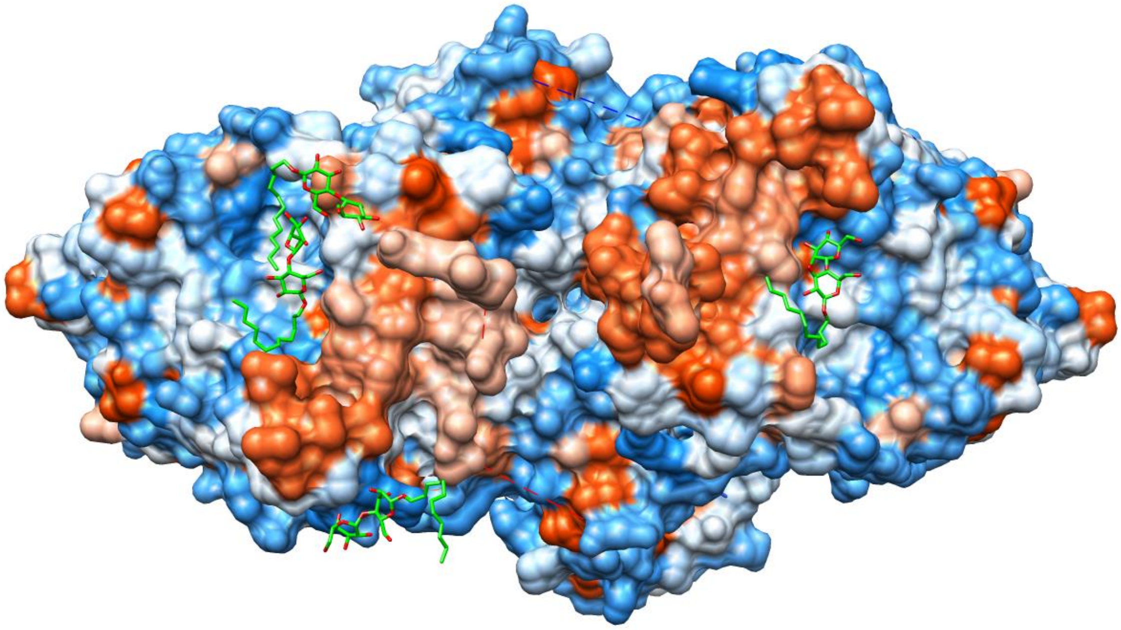

Figure S6. Charged surface distribution for the crystallographic dimer of AncFMO1. Distribution of charge around the surface of AncFMO1, with orange, white and blue representing hydrophobic, neutral and hydrophilic residues, respectively. Darker and lighter colors portray the extent of the characteristic, with the former and latter representing higher or lower, respectively. Large parallel hydrophobic strips across the bottom of the dimer are visible (dark orange), lined by a ring of positively polar residues (dark blue). Molecules of detergent, DDM are shown clustering around these hydrophobic strips in green.

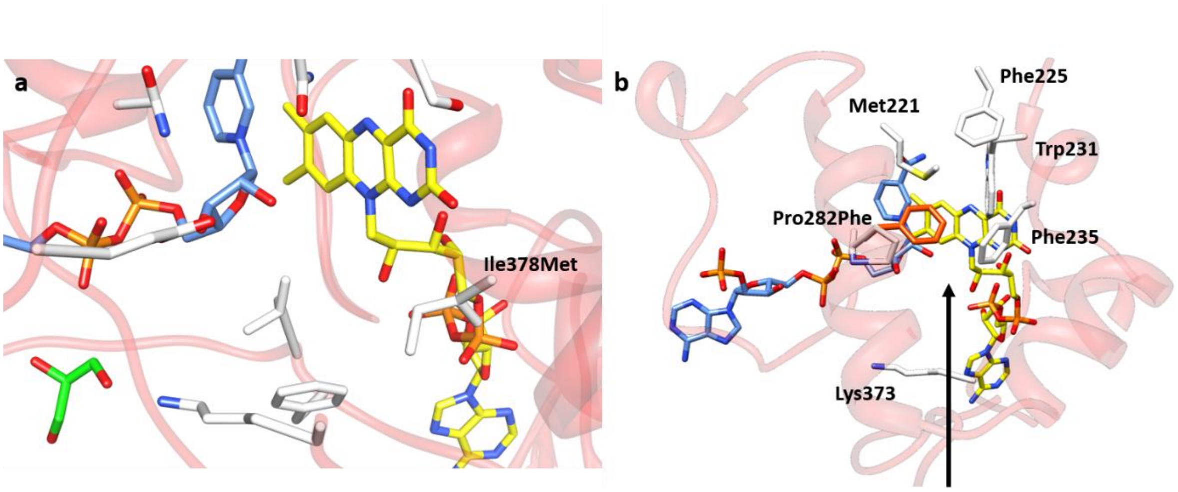

Figure S7. Key residue changes between AncFMO1 and hFMO1. a, The active site of AncFMO1 is shown with emphasis directed towards Ile378. This site is a methionine residue in hFMO1. **b**, The trajectory of the hydrophobic substrates with respect to the α-helical triad is shown by a black arrow. FAD, $NADP^+$, Glycerol, AncFMO1 residues and theoretical hFMO1 residues are shown in yellow, cornflower blue, green, white and orange, respectively.

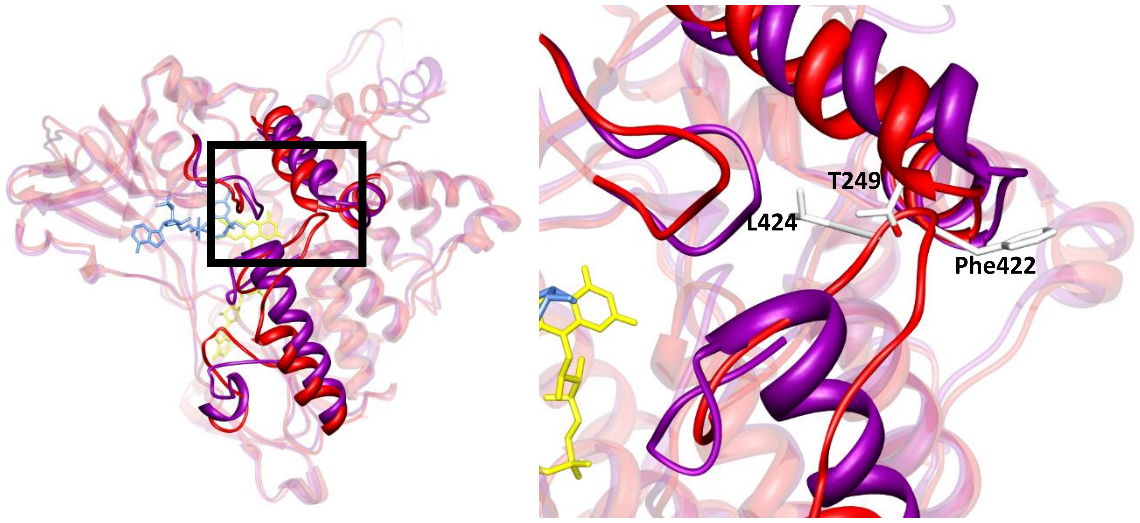

Figure S8. Overarching loop. The AncFMO1 monomer (red) is superimposed with the monomer of AncFMO3-6 (dark magenta). The unique loop feature exhibited by AncFMO1 is shown in the expanded black boxes. FAD, $NADP^+$ and residue sidechains are shown in yellow, cornflower blue and white, respectively.

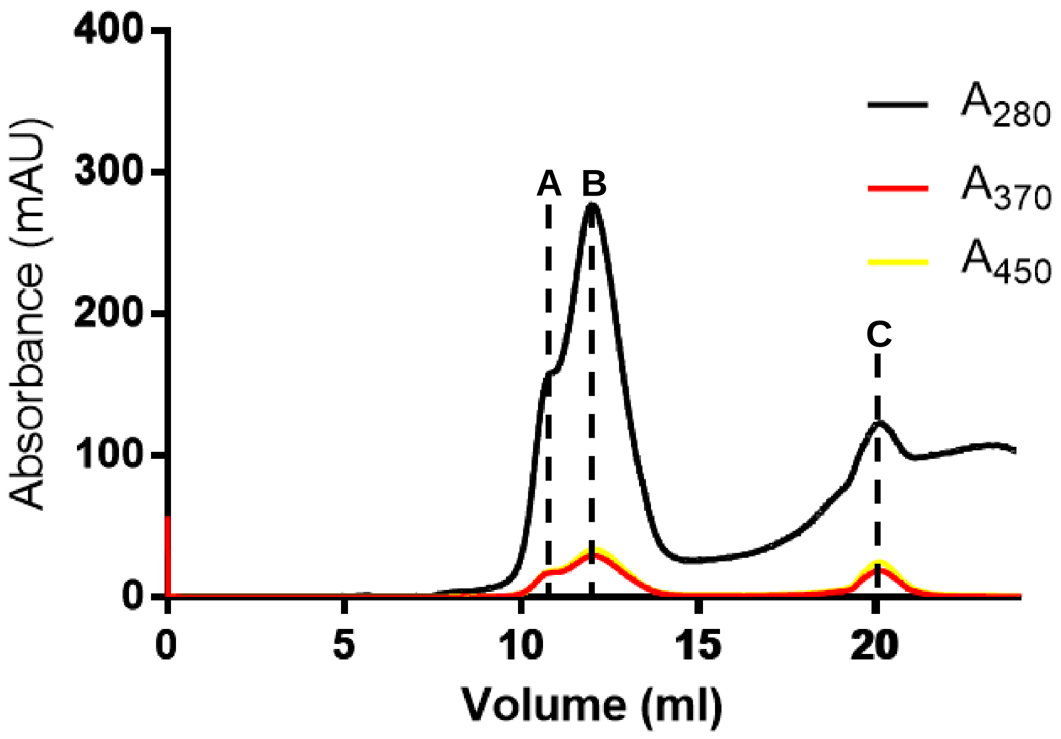

Figure S9. Gel filtration chromatogram of AncFMO1. Previously characterized AncFMOs eluted in a bell-shaped manner between 10.5 and 11 mL(1), as a monodisperse peak. AncFMO1 exhibits two elution volumes at 10.8 and 12 mL represented by peaks A and B respectively. These two peaks overlap with, A, acting as a shoulder to the more pronounced peak, B. These two peaks collectively represent two different oligomeric states of AncFMO1. Crystallographic studies were performed with protein derived from both peaks, A and B, concentrated down together with the hypothesis that the oligomeric states A and B (derived from peaks A and B, respectively) were in dynamic equilibrium between each other. With the previously crystallized AncFMOs crystallizing whilst exhibiting the oligomeric state, A, we postulated that the oligomer B would shift to the oligomeric state A as that oligomer precipitates out of solution in crystalline form. Traces in black, red and yellow represent wavelengths 280, 370 and 450 nm, respectively, measured in absorbance, mAU. Band C corresponds to the excess of FAD added to the protein solution before size exclusion chromatography.

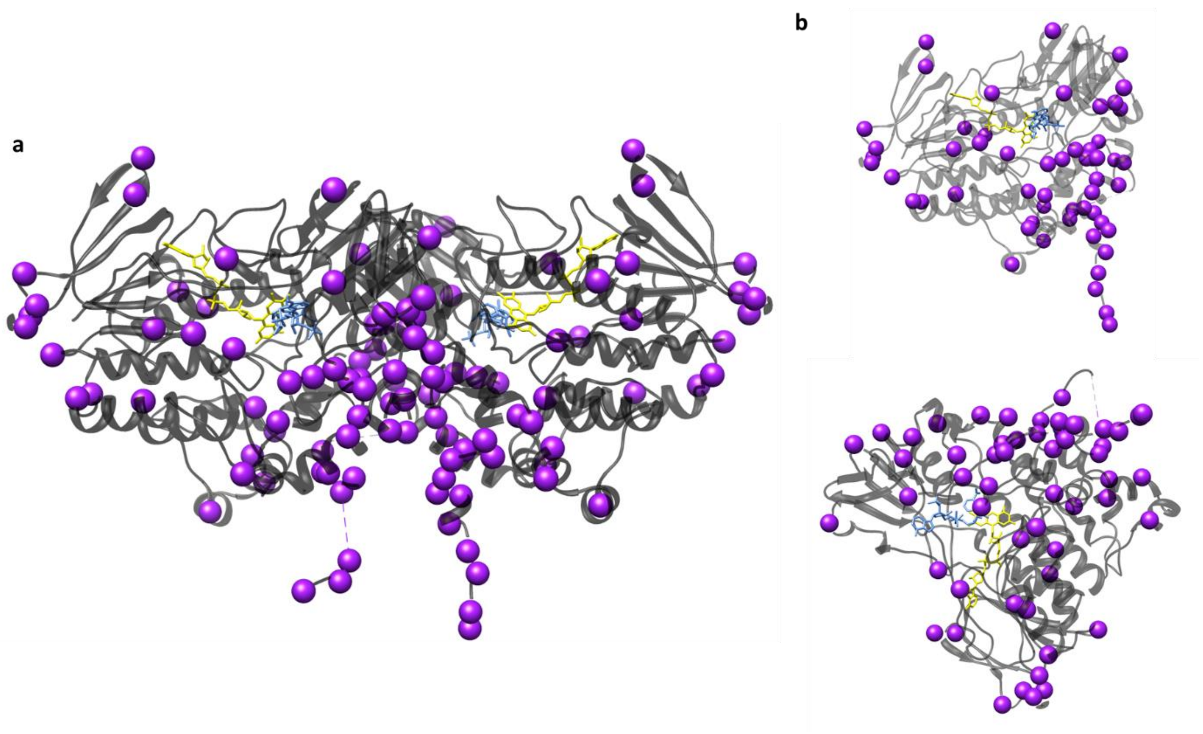

Figure S10. Differing residues between AncFMO1 and hFMO1. a, The differences between AncFMO1 and hFMO1 with respect to the crystallographic dimer are shown with purple spheres. AncFMO1 and hFMO1 share a percentage sequence identity and percentage sequence similarity of 89.9% (54 residues) and 94.2% (29 residues), respectively, demonstrating the extensive likeness between the two enzymes. **b,** The differences observed with respect to monomer in two different orientations. The FAD and NADP molecules are shown in yellow and cornflower blue, respectively.

AncFMO1

>AncFMO1m

MAKRVAIVGAGVSGLASIKCCLEEGLEPTCFERSDDLGGLWRFTEHVEEGRASLYKSVVSNSCKEMSCYSDFPFP
EDYPNYVPNSQFLEYLKMYANRFNLLKHIQFKTKVCSVTKCPDFTVTGQWEVVTQHEGKQESAIFDAVMVCTGFL
TDPYLPLDSFPGINTFKGQYFHSRQYKHPDIFKDKRVLVVGMGNSGTDIAVEASHLAKKVFLSTTGGAWVMSRVF
DSGYPWDMVFTTRFQNMLRNSLPTPIVTWLMARKMNSWFNHANYGLVPEDRTQLREPVLNDELPGCIITGKVLIK
PSIKEVKENSVIFNNTPKEEPIDIIVFATGYTFAFPFLDESVVKVENGQASLYKYIFPAHLPKPTLAVIGLIKPL
GSIIPTGETQARWAVRVLKGINKLPPQSVMIEEVNARKENKPSGFGLCYCKALQSDYITYIDELLTYINAKPNLL
SMLLTDPRLALTIFFGPCTPYQFRLTGPGKWEGARNAILTQWDRTFKVTKTRIVQESPSPFASLLKLLSLPVLLL
ALLLMC

site	ML-Anc		Alt-Anc	
	State	PP	Alt state	PP
152	D	0.51	N	0.49
190	V	0.62	I	0.38
221	M	0.55	I	0.45
236	T	0.64	M	0.36
243	L	0.78	F	0.21
253	T	0.52	N	0.48
256	M	0.74	L	0.22
389	A	0.59	V	0.41
442	Y	0.54	S	0.41
450	L	0.72	F	0.27

AncFMO4

>AncFMO4m

MAKRVAVIGAGVSGLSSIKCCLDEDLEPTCFERSDDFGGLWKFTESSKDGMTRVYRSLVTNVCKEMSCYSDFPFQ
EDYPNFMNHAKFWDYLREFAEHFDLLKYIQFKTTVCSVTKCPDFSETGQWDVVTETEGKQDRAVFDAVMVCTGHF
LNPHLPLESFPGIHKFKGQILHSQEYRTPEAFQGKRVLVIGLGNTGGDIAVELSRTAAQVFLSTRTGTWVLSRSS
DGGYPFNMMTTRRCHNFIAQVLPSCFLNWIQERQMNKRFNHENYGLSITKGKKKKAIVNDELPTCILCGTVTVKT
SVKEFTETSAVFEDGTVEANIDVVIFTTGYTFSFPFLEEPLKSLCTKKIFLYKRVFPPNLEKATLAIIGLISLTG
SILAGTELQARWATRVFKGLCKIPPSQKLMAEVTKKEQLIKRGVIKDTSQDKLDYISYMDELAACIGAKPNIPLL
FLKDPRLAWEVFFGPCTPYQYRLVGPGKWDGARNAILTQWDRTLKPLKTRIVADSSKPASMSHYLKVWGAPVLLA
SLLLICKSSLFLKLVRDKLQDRISPYLISLWRGS

site	ML-Anc		Alt-Anc	
	State	PP	State	PP
37	F	0.76	I	0.24
116	C	0.70	R	0.30
177	R	0.76	K	0.24
221	L	0.54	I	0.24
231	F	0.51	W	0.30
235	T	0.38	V	0.36
240	H	0.78	N	0.20
319	A	0.63	E	0.37
349	I	0.66	M	0.32
354	R	0.62	Q	0.29
363	A	0.54	T	0.36
409	T	0.63	V	0.33
474	V	0.54	M	0.40
494	L	0.71	M	0.27
503	A	0.51	P	0.45
507	K	0.64	Q	0.34
522	V	0.68	I	0.32
524	L	0.64	F	0.23
541	R	0.58	Q	0.33
552	L	0.48	R	0.39
553	I	0.49	V	0.42
555	L	0.70	I	0.26
557	R	0.62	Q	0.25
558	G	0.73	N	0.21

Data S1. AncFMO sequences and lists of ambiguously reconstructed sites.

Accession code	Organism	Taxonomic rank	FMO clade
L5LMT2	*Myotis davidii*	Mammalia	**FMO3/6**
W5PRR0	*Ovis aries*		
Q8HYJ9	*Bos taurus*		
L8I8K7	*Bos mutus*		
A0A140C4U0	*Bubalus bubalis*		
W5PRR0	*Ovis aries*		
XP_005690690	*Capra hircus*		
XP_020742463	*Odocoileus virginianus texanus*		
XP_007165360	*Balaenoptera acutorostrata scammoni*		
XP_004269798	*Orcinus orca*		
XP_019807672	*Tursiops truncatus*		
XP_022415770	*Delphinapterus leucas*		
XP_007113108	*Physeter catodon*		
S9YQ93	*Camelus ferus*		
XP_010955230	*Camelus bactrianus*		
XP_006211173	*Vicugna pacos*		
A0A1R7SPQ2	*Sus crofa*		
Q95LA1	*Canis lupus*		
M3YVX5	*Mustela putorious*		
XP_004408956	*Odobenus rosmarus*		
XP_021538316	*Neomonachus schauinslandi*		
XP_008694265	*Ursus maritimus*		
M3VZK1	*Felis catus*		
XP_007088793	*Panthera tigris*		
XP_014917803	*Acinonyx jubatus*		
XP_012513247	*Propithecus coquereli*		
XP_012620619	*Microcebus murinus*		
XP_004688531	*Condylura cristata*		
XP_006147860	*Tupaia chinensis*		
A0A1U7TTF6	*Tarsius syrichta*		
H0XFF2	*Otolemur garnettii*		
F6QL08	*Equus caballus*		
XP_014700658	*Equus asinus*		
F7HH82	*Callithrix jacchus*		
Q5REM1	*Pongo abelii*		
Q8SPQ7	*Macaca mulatta*		
A0A0D9RGQ9	*Chlorocebus sabaeus*		
A0A023JCA1	*Macaca fascicularis*		
Q53FW5 (FMO3)	*Homo sapiens*		
A0A0G2JSI0	*Rattus norvegicus*		
A0A1U7Q764	*Mesocricetus auratus*		
XP_021054533	*Mus pahari*		
XP_005364035	*Microtus ochrogaster*		
XP_015849094	*Peromyscus maniculatus bairdii*		
XP_008846748	*Nannospalax galili*		

XP_020025626	*Castor canadensis*		
XP_006887483	*Elephantulus edwardii*		
U3KLZ1	*Oryctolagus cuniculus*		
XP_004853513	*Heterocephalus glaber*		
XP_004706887	*Echinops telfairi*		
XP_010835019	*Bison bison*		
W5PS24	*Ovis aries*		
XP_005216983	*Bos taurus*		
XP_004013737	*Ovis aries*		
XP_005690691	*Capra hircus*		
XP_005902457	*Bos mutus*		
XP_006075483	*Bubalus bubalis*		
I3LIW4	*Sus crofa*		
XP_006211175	*Vicugna pacos*		
XP_006173292	*Camelus ferus*		
J9P0F0	*Canis lupus*		
M3W9K9	*Felis catus*		
XP_019280198	*Panthera pardus*		
M3YVX8	*Mustela putorious*		
XP_022364137	*Enhydra lutris*		
XP_019664755	*Ailuropoda melanoleuca*		
XP_008586856	*Galeopterus variegatus*		
G7MFB5	*Macaca mulatta*		
G8F2N3	*Macaca fascicularis*		
XP_011744241	*Macaca nemestrina*		
XP_007987722	*Chlorocebus sabaeus*		
XP_017722368	*Rhinopithecus bieti*		
XP_524962	*Pan troglodytes*		
O60774 (FMO6)	*Homo sapiens*		
G1TFY5	*Oryctolagus cuniculus*		
XP_004454775	*Dasypus novemcinctus*		
XP_006887525	*Elephantulus edwardii*		
XP_004706886	*Echinops telfairi*		
XP_006872703	*Chrysochloris asiatica*		
M0R553	*Rattus norvegicus*		
A0A1U7Q721	*Mesocricetus auratus*		
XP_021054447	*Mus pahari*		
XP_003505573	*Cricetulus griseus*		
XP_006974814	*Peromyscus maniculatus bairdii*		
XP_008846747	*Nannospalax galili*		
XP_007525282	*Erinaceus europaeus*		
XP_004688176	*Condylura cristata*		
I3M344	*Ictidomys tridecemlinea*		
XP_015347117	*Marmota marmota*		
XP_006147858	*Tupaia chinensis*		
XP_008144061	*Eptesicus fuscus*		

XP_005867703	*Myotis brandtii*		
G3SQL7	*Loxodonta africana*		
XP_012786396	*Ochotona princeps*		
A0A093JJ61	*Eurypyga helias*	Aves	
A0A0A0B1A6	*Charadrius vociferus*		
A0A087VQN0	*Balearica regulorum*		
A0A091XN66	*Opisthocomus hoazin*		
A0A091R3N8	*Leptosomus discolor*		
A0A091JN32	*Egretta garzetta*		
A0A091G0C9	*Cuculus canorus*		
A0A091TAH2	*Phaethon lepturus*		
A0A093JBD9	*Fulmarus glacialis*		
A0A091KMX8	*Chlamydotis macqueenii*		
A0A091MAZ1	*Cariama cristata*		
A0A093CLM1	*Pterocles gutturali*		
U3I4R2	*Anas platyrhynchos*		
Q8QH01	*Gallus gallus*		
K9UTG7	*Coturnix coturnix*		
A0A099ZAQ2	*Tinamus guttatus*		
M7C297	*Chelonia mydas*	Testudines	
A0A1L8GN41	*Xenopus laevis*	Amphibia	
F7CV72	*Xenopus tropicalis*		
XP_005245102	*Homo sapiens*	Mammalia	**FMO4**
A0A2R8ZJU8	*Pan paniscus*		
H2Q0L6	*Pan troglodytes*		
Q5RDN6	*Pongo abelii*		
G1RYU9	*Nomascus leucogenys*		
A0A2K5IYC3	*Colobus angolensis palliatus*		
A0A2K6PMQ1	*Rhinopitecus roxellana*		
A0A2K6KY70	*Rhinopitecus bieti*		
A0A2K5ZU18	*Mandrillus leucophaeus*		
A0A2K5LB79	*Cercocebus atys*		
F7D7C4	*Macaca mulatta*		
G8F2N5	*Macaca fascicularis*		
A0A2K6EBX8	*Macaca nemestrina*		
A0A0D9RGS9	*Chlorocebus sabaeus*		
G3RA17	*Gorilla gorilla*		
A0A2K6V3P4	*Saimiri boliviensis*		
A0A2K5DLW2	*Aotus nancymaae*		
A0A2K5R6I3	*Cebus capucinus*		
F7I9S6	*Callithrix jacchus*		
A0A2K6V3M8	*Saimiri boliviensis*		
XP_012042660	*Ovis aries*		
L8I7Q9	*Bos mutus*		
G5E5J8	*Bos taurus*		
W5PT88	*Ovis aries*		

A0A2Y9FGD8	*Physeter catodon*		
A0A2Y9M166	*Delphinapterus leucas*		
A0A2U3ZYV0	*Tursiops truncatus*		
F1S6B7	*Sus crofa*		
G1MF53	*Ailuropoda melanoleuca*		
M3WH62	*Felis catus*		
A0A2Y9GGS8	*Neomonachus schauinslandi*		
A0A2U3ZX57	*Odobenus rosmarus*		
A0A2Y9JTV	*Enhydra lutris*		
M3YVY4	*Mustela putorious*		
E2RI71	*Canis lupus*		
F6ZTZ1	*Equus caballus*		
A0A2K6GCQ4	*Propithecus coquereli*		
H0XFG2	*Otolemur garnettii*		
A0A1U7U321	*Tarsius syrichta*		
P36367	*Oryctolagus cuniculus*		
A0A2Y9RG03	*Trichechus manatus*		
G3SX00	*Loxodonta africana*		
H0VQ15	*Cavia porcellus*		
G5BGT2	*Heterocephalus glaber*		
Q8VHG0	*Mus musculus*		
Q8K4B7	*Rattus norvegicus*		
A0A1U8BUR7	*Mesocricetus auratus*		
L9KV99	*Tupaia chinensis*		
A0A1S2ZVI0	*Erinaceus europaeus*		
A0A1V4JY45	*Patagioneas fasciatus*	Aves	
A0A093FGE7	*Gavia stellata*		
U3I700	*Anas platyrhynchos*		
M7BBI8	*Chelonia mydas*	Testudines	
K7FTQ9	*Pelodiscus sinensis*		
NP_036924	*Rattus norvegicus*	Mammalia	**FMO1**
NP_002012	*Homo sapiens*		
A0A2R9CCZ4	*Pan paniscus*		
A0A2J8LLM7	*Pan troglodytes*		
G1RYT9	*Nomascus leucogenys*		
H2N4Q7	*Pongo abelii*		
G3R5S9	*Gorilla gorilla*		
A0A2K6KT99	*Rhinopithecus bieti*		
A0A2K6RMJ0	*Rhinopitecus roxellana*		
A0A2K5JQS5	*Colobus angolensis palliatus*		
A0A2K6E4Q2	*Macaca nemestrina*		
G8F2N4	*Macaca fascicularis*		
F7CKS4	*Macaca mulatta*		
A0A2K5XVP0	*Mandrillus leucophaeus*		
A0A2K5LI13	*Cercocebus atys*		
A0A0D9RGS2	*Chlorocebus sabaeus*		

A0A2K6S2A8	*Saimiri boliviensis*		
A0A2K5DNS9	*Aotus nancymaae*		
F6TF83	*Callithrix jacchus*		
A0A2K6GJC5	*Propithecus coquereli*		
H0XFG0	*Otolemur garnettii*		
M3YVY3	*Mustela putorious*		
A0A2Y9JX65	*Enhydra lutris*		
Q95LA2	*Canis lupus*		
D2I021	*Ailuropoda melanoleuca*		
A0A2U3XYA5	*Leptonychotes weddellii*		
M3W9L4	*Felis catus*		
P16549	*Sus crofa*		
G5E5R0	*Bos taurus*		
W5PSR9	*Ovis aries*		
L9KVX0	*Tupaia chinensis*		
A0A1U7U1X5	*Tarsius syrichta*		
A0A1S2ZVI9	*Erinaceus europaeus*		
L5LP31	*Myotis davidii*		
L7N1I9	*Myotis lucifugus*		
P17636	*Oryctolagus cuniculus*		
G3TPA3	*Loxodonta africana*		
A0A218V4N6	*Lonchura striata*	Aves	
A0A091LMM3	*Chlamydotis macqueenii*		
A0A0A0B033	*Charadrius vociferus*		
A0A091JJZ4	*Egretta garzetta*		
A0A091KNM4	*Colius striatus*		
A0A091QCB7	*Merops nubicus*		
A0A093PIP7	*Manacus vitellinus*		
A0A091G2R8	*Cuculus canorus*		
K7FK79	*Pelodiscus sinensis*	Testudines	
Q5REK0	*Pongo abelii*	Mammalia	**FMO2**
G1RYT1	*Nomascus leucogenys*		
Q28505	*Macaca mulatta*		
A0A023JBW5	*Macaca fascicularis*		
F7FJA6	*Callithirx jacchus*		
XP_007987720	*Chlorocebus sabaeus*		
XP_011826204	*Mandrillus leucophaeus*		
XP_017722374	*Rhinopithecus bieti*		
NP_001009008	*Pan troglodytes*		
NP_001451	*Homo sapiens*		
XP_004027940	*Gorilla gorilla*		
XP_010334438	*Saimiri boliviensis*		
XP_021523737	*Aotus nancymaae*		
XP_017389638	*Cebus capucinus*		
M3YVY2	*Mustela putorious*		
XP_022363881	*Enhydra lutris*		

G1MF96	*Ailuropoda melanoleuca*		
XP_008694286	*Ursus maritimus*		
XP_021538314	*Neomonachus schauinslandi*		
E2RHC8	*Canis lupus*		
F6T988	*Equus caballus*		
XP_014700659	*Equus asinus*		
XP_014638919	*Ceratotherium simum simum*		
XP_006872704	*Chrysochloris asiatica*		
XP_006887484	*Elephantulus edwardii*		
XP_004454778	*Dasypus novemcinctus*		
A0A1U7QFV1	*Mesocricetus auratus*		
XP_003505578	*Cricetulus griseus*		
XP_006974813	*Peromyscus maniculatus bairdii*		
G3V6F6	*Rattus norvegicus*		
Q8K2I3	*Mus musculus*		
XP_021054156	*Mus pahari*		
XP_020025620	*Castor canadensis*		
XP_008846746	*Nannospalax galili*		
XP_003130152	*Sus crofa*		
XP_007165362	*Balaenoptera acutorostrata scammoni*		
XP_010955205	*Camelus bactrianus*		
W5PS98	*Ovis aries*		
G5E540	*Bos taurus*		
XP_005690692	*Capra hircus*		
XP_010850196	*Bison bison*		
XP_006075480	*Bubalus bubalis*		
XP_020759823	*Odocoileus virginianus texanus*		
G1SND1	*Oryctolagus cuniculus*		
G3TH74	*Loxodonta africana*		
XP_012658178	*Otolemur garnettii*		
XP_019503343	*Hipposideros armiger*		
S7MVT1	*Myotis brandtii*		
A0A0P6JH69	*Heterocephalus glaber*		
A0A091DJC6	*Fukomys damarensis*		
P36366	*Cavia porcellus*		
M3W9L1	*Felis catus*		
XP_007088786	*Panthera tigris*		
XP_019280219	*Panthera pardus*		
XP_019601297	*Rhinolophus sinicus*		
XP_005374888	*Chinchilla lanigera*		
XP_015347321	*Marmota marmota*		
L9KUM1	*Tupaia chinensis*		
K7FEU6	*Pelodiscus sinensis*	Testudines	
M7BR11	*Chelonia mydas*		
Q6DF15	*Xenopus tropicalis*	Amphibia	N/A
Q6PA74	*Xenopus laevis*		

A0A1L8GN32	*Xenopus laevis*		
F6Y350	*Xenopus tropicalis*		
A0A1L8GN11	*Xenopus laevis*		
Q5BKK2	*Xenopus tropicalis*		
F6TLP5	*Xenopus tropicalis*		
F6TKY1	*Xenopus tropicalis*		
NP_001075714	*Oryctolagus cuniculus*		
W5QH53	*Ovis aries*		
A6QLN7	*Bos taurus*		
NP_001452	*Homo sapiens*		
H2PZU9	*Pan troglodytes*		
G3S9Y3	*Gorilla gorilla*		
G1QH17	*Nomascus leucogenys*		
A0A2K5NFE4	*Cercocebus atys*		
A0A096NWS4	*Papio anubis*		
G7NTQ5	*Macaca fascicularis*		
A0A1D5Q215	*Macaca mulatta*		
A0A0D9S0K5	*Chlorocebus sabaeus*		
XP_010382298	*Rhinopithecus roxellana*		
XP_011806642	*Colobus angolensis palliatus*		
H2N639	*Pongo abelii*		
F7ALU6	*Callithrix jacchus*		
XP_012327405	*Aotus nancymaae*		
H0X7S2	*Otolemur garnettii*		
A0A1U7TPN0	*Tarsius syrichta*		
E2QUP4	*Canis lupus*		
XP_023114833	*Felis catus*	Mammalia	**FMO5**
XP_007092639	*Panthera tigris*		
XP_014939496	*Acinonyx jubatus*		
XP_007182187	*Balaenoptera acutorostrata scammoni*		
XP_004285858	*Orcinus orca*		
XP_023975388	*Physeter catodon*		
G1QEL7	*Myotis lucifugus*		
S7MVU6	*Myotis brandtii*		
F1SDB7	*Sus crofa*		
F6PLR9	*Equus caballus*		
G3T364	*Loxodonta africana*		
XP_006900388	*Elephantulus edwardii*		
I3MPC8	*Ictidomys tridecemlinea*		
P49109	*Cavia porcellus*		
G5BA06	*Heterocephalus glaber*		
XP_005413529	*Chinchilla lanigera*		
XP_004646243	*Octodon degus*		
A0A091EEE1	*Fukomys damarensis*		
P97872	*Mus musculus*		
Q8K4C0	*Rattus norvegicus*		
A0A1A6H802	*Neotoma lepida*		
G3HP87	*Cricetulus griseus*		

S9XQV9	*Camelus ferus*		
A0A1S3FCC7	*Dipodomys ordii*		
L5K3R4	*Pteropus alecto*		
F7FCD2	*Monodelphis domestica*		
G3WY85	*Sarcophilus harris*		
A0A1S3A3I9	*Erinaceus europaeus*		
XP_004589355	*Ochotona princeps*		
XP_004651790	*Jaculus jaucus*		
XP_026633339	*Microtus ochrogaster*		
E1C7G8	*Gallus gallus*	Aves	
A0A091EER2	*Corvus brachyrhynchos*		
H1A3R9	*Taeniopygia guttata*		
U3JME7	*Ficedula albicollis*		
A0A091UWC8	*Nipponia nippon*		
A0A087QQJ0	*Aptenodytes forster*		
A0A091GDX2	*Cuculus canorus*		
A0A091I0D0	*Calypte anna*		
G1MZ19	*Meleagris gallopavp*		
A0A093NXE8	*Pygoscelis adeliae*		
U3J9N7	*Anas platyrhynchos*		
M7C2B2	*Chelonia mydas*	Testudines	
M7BR25	*Chelonia*		
M7B995	*Chelonia*		
K7G1T3	*Pelodiscus sinensis*		
F6T1Q3	*Xenoupus tropicalis*	Amphibia	
Q6PB07	*Xenopus laevis*		
Q6AX90	*Xenopus laevis*		
F6T1R3	*Xenoupus tropicalis*		
A0A1L8GN28	*Xenopus laevis*		
F6S648	*Xenopus tropicalis*		
A0A1L8GN11	*Xenopus laevis*		
A0A1L8GN1	*Xenopus laevis*		
Q4SLV0	*Tetraodon nigroviridis*	Actinopterygii/ Actinopteri	N/A (root)
A0A0F8AHM2	*Larimichthys crocea*		
A0A2U9C3W1	*Scophthalmus maximus*		
H3A7E3	*Latimeria chalumnae*	Sarcopterygii/ Coelacanthimorpha	
V9KV64	*Callorhinchus milii*	Chondrichthyes	
V9KI45	*Callorhinchus milii*		
C3ZTI4	*Branchiostoma floridae*	Cephalochordata	

Table S1. Jawed vertebrates FMOs dataset. The protein accession codes (Genbank or Uniprot) are given for each of the sequences collected with the corresponding species names. The taxonomic rank is given as the class or order (for terrestrial tetrapods) and superclass/class or phylum (for the root sequences). The FMO paralog clade was assigned for each sequence based on the phylogeny and the sequences from *Homo sapiens* are written in red for reference. N/A: not assigned. The two studied FMO paralog clades FMO1 and FMO4 are highlighted in lime in agreement with the coloring of the phylogeny.

**protein**	**melting temperature (T_m) / °C**	
	no $NADP^+$	200 µM $NADP^+$
**AncFMO1**	47	52
**AncFMO2**(1)	53	70
**AncFMO3-6**(1)	60	66.5
**AncFMO5**(1)	55	59
**hFMO3**[a]	44.5	44.5
**hFMO5**(1)	49	48.5

Table S2. Melting temperatures of the AncFMOs. Melting temperatures were measured in triplicate by using the ThermoFAD technique for AncFMOs 2, 3-6 and 5, and the TychoTMNT.6 system for AncFMO1 (see materials and methods). [a]hFMO3 melting temperature in Triton X-100 reduced (personal communication F. Fiorentini).

type of oxidation	Reaction	conversion[a]	*ee* (%)[b]
N-oxidation	Benzydamine → Benzydamine N-oxide<br>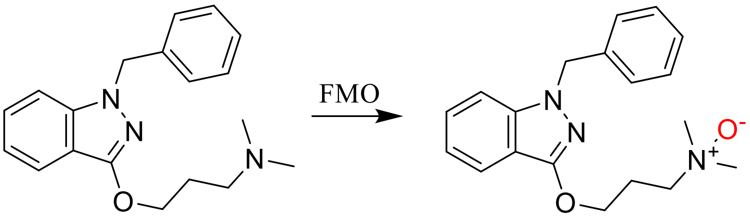	>99%	n.a.
	Tamoxifen → Tamoxifen N-oxide<br>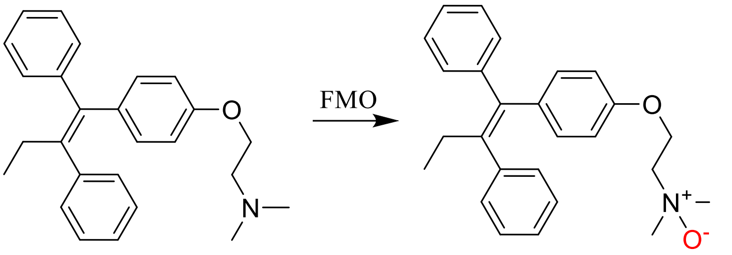	>99%	n.a.
S-oxidation	Thioanisole → Thioanisole sulfoxide<br>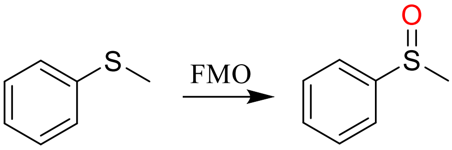	>99%	96 (*R*)
B-V oxidation	bicyclo[3.2.0]hept-2-en-6-one<br>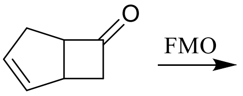<br>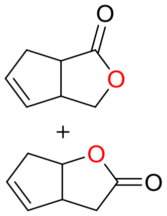<br>3-oxabicyclo[3.3.0]oct-6-en-2-one + 2-oxabicyclo[3.3.0]oct-6-en-3-one	40%	n.d.

Table S3. Conversions catalyzed by AncFMO1. Reactions were incubated at 30 °C for 18 hours, at pH 7.5 in the presence of a NADPH recycling system. [a] Conversion of Baeyer-Villiger substrates and thioanisole were measured on GC-MS while N-oxides were identified on HPLC. [b] Enantiomeric excess was measured by HPLC. n.a.: not applicable, n.d.: not determined.

1. Nicoll, C. R., Bailleul, G., Fiorentini, F., Mascotti, M. L., Fraaije, M. W., and Mattevi, A. (2020) Ancestral-sequence reconstruction unveils the structural basis of function in mammalian
